# The working life expectancy of American adults experiencing depression

**DOI:** 10.1007/s00127-023-02547-4

**Published:** 2023-09-07

**Authors:** Kathleen G. Dobson, Monique A. M. Gignac, Cameron A. Mustard

**Affiliations:** 1 Institute for Work and Health, Toronto, Canada; 2 Dalla Lana School of Public Health, University of Toronto, Toronto, Canada

**Keywords:** Depression, Employment, Mental illness, Working life expectancy, Unemployment, Growth mixture modeling, Multistate modeling, Markov model

## Abstract

**Objectives:**

To estimate the working life expectancies (WLE) of men and women with depression, examining depression by symptom trajectories from the late 20s to early 50s, and to estimate WLE by race/ethnicity and educational attainment.

**Methods:**

Data from 9206 participants collected from 1979 to 2018 in the US National Longitudinal Survey of Youth 1979 cohort were used. Depression was measured using the *Center for Epidemiologic Studies Depression Scale Short Form* at four time points (age 28–35, age 30–37, age 40, and age 50). Labor force status was measured monthly starting at age 30 until age 58–62. Depressive symptom trajectories were estimated using growth mixture modeling and multistate modeling estimated WLE from age 30–60 for each gender and depressive symptom trajectory.

**Results:**

Five latent symptom trajectories were established: a persistent low symptom trajectory (*n* = 6838), an episodic trajectory with high symptoms occurring before age 40 (*n* = 995), an episodic trajectory with high symptoms occurring around age 40 (*n* = 526), a trajectory with high symptoms occurring around age 50 (*n* = 570), and a persistent high symptom trajectory (*n* = 277). The WLE for men at age 30 was 30.3 years for the persistent low symptom trajectory, 22.8 years for the episodic before 40 trajectory, 19.6 years for the episodic around age 40 trajectory, 18.6 years for the episodic around age 50 trajectory, and 13.2 years for the persistent high symptom trajectory. Results were similar for women. WLE disparities between depression trajectories grew when stratified by race/ethnicity and education level.

**Conclusions:**

Roughly a quarter of individuals experienced episodic depressive symptoms. However, despite periods of low depressive symptoms, individuals were expected to be employed ~5–17 years less at age 30 compared to those with low symptoms. Accessible employment and mental health disability support policies and programs across the working life course may be effective in maintaining work attachment and improving WLE among those who experience depression.

**Supplementary Information:**

The online version contains supplementary material available at 10.1007/s00127-023-02547-4.

## Introduction

Depression is a major health and economic challenge. Prior to the COVID-19 pandemic, the point prevalence of depression among American adults was 6.6%, and 11.0% for either depression or an anxiety disorder [1]. The economic burden of major depressive disorder in the United States has been estimated to be $326.2 billion USD, with 61% of this cost attributed to work productivity losses [2]. Despite improvements in the treatment of depression—which have increased employment retention—individuals with depression still retain lower likelihoods of stable employment [3], upward career progression [4], and increased risk of unemployment [5] and premature labor force exit [6].

Quantifying the labor market burden of depression is critical to inform welfare, health, and social services policy [7, 8]. Currently, there is a lack of research quantifying working life expectancy (WLE): the average number of years an individual is expected to participate in employment until they permanently exit the labor force.^9^ In 2019, Pedersen and colleagues found that WLE at age 40 was 14.6 years for Danish women exhibiting depressive symptoms (3.3 years lower vs. those with no depression) and 17.3 years for men exhibiting depressive symptoms (vs. 19.6 years). Plana-Ripoll and colleagues (2022) found that Danish civil servants diagnosed with a mood disorder lost 10.3 years of working life when compared to the general Danish population and studied over a 40-year period. In both studies, respondents were categorized as experiencing depression at one time point, limiting understanding about how the episodic nature of depression may influence WLE. Although depression is clinically recognized as episodic, few studies have estimated the longitudinal trajectories of depressive episodes and examined their impact on employment [3].

A second gap in the understanding of the WLE among those experiencing depression is the absence of information examining race/ethnicity and education levels. These factors may influence risk of depression [9], symptom severity [10], and influence labor market experiences [9, 11–14]. Higher education is significantly associated with favorable employment outcomes among those experiencing depression [15]. Moreover, studies exploring differences in WLE between White and Black individuals have found that racialized differences are greater among men than women, and that these differences diminish with age [16].

## Objectives

Understanding the transitions from labor force entry to exit for individuals experiencing depression is critical knowledge for policy makers to create policies focused on employment sustainability and return to work. Estimating WLE can uncover transition patterns between employment, unemployment, and labor force non-participation. For example, it is unclear whether someone who has a depressive episode early in their career can “catch up” to the employment experience of someone who does not experience depression, or whether an established career can help buffer the impacts of experiencing a depressive episode on employment in later adulthood. Therefore, the purpose of this study was to define depressive symptom trajectories experienced by American men and women from age 28 to 50, and to calculate the WLE for men and women between the age of 30 and 64 with diverse depressive symptom trajectories, race/ethnicities, and educational levels at age 30.

## Methods

### Sample

We used data from the National Longitudinal Survey of Youth 1979* (*NLSY79) cohort, which collects information on the working lives of a cohort of 12,686 Americans born between 1957 and 1964 [17, 18]. First interviews occurred in 1979 when participants were 14–21 years old [18]. Surveys have been completed annually or biannually, with the last survey cycle completed in 2018 when participants were 54–62 years old.

The NLSY79 sample aimed to be a representative of all youth born between 1957 and 1964 [17]. The overall sample included a cross-sectional subsample (*n* = 6111) representing non-institutionalized civilian youth in the United States; a supplemental subsample (*n* = 5295) which oversampled Hispanic/Latino, Black, and economically disadvantaged Non-Hispanic, Non-Black (NBNH) youth; and a subsample of youth (*n* = 1280) serving in the military. Of these participants, 6403 were men, 7510 were NBNH, 3174 were Black, and 2002 were Hispanic/Latino. The full military subsample was no longer eligible for interviewing after 1984; however, 201 participants were randomly selected to remain in the survey. After 1990, NBNH respondents from the supplemental subsample were no longer interviewed [19].

Retention and response rates were over 90% between 1979 and 1993, over 80% between 1994 and 2008, and close to 80% between 2008 and 2018 [19]. Participants were eligible to participate at each survey cycle, regardless of whether they were surveyed at the previous cycle. Approximately 45% of individuals eligible for this study completed all survey cycles and 22% completed 25–27 cycles, providing excellent information on participants’ life histories.

The present study focused on civilian working-age individuals (age 18–62 years) with information on labor force status and depression measures (Supplemental Figure 1.1). We excluded those in the military and economically disadvantaged NBNH participant subsamples (*n* = 2923), as these groups were no longer surveyed after 1990 [19]. We also excluded participants who had died prior to 1993 (*n* = 152), and 405 individuals with less than one depressive symptom measurement. These individuals were generally older in 1979, men, and had missed 6 or more survey cycles. Thus, the analytic sample was 9206 participants.

### Study measures

In each cycle, participants were asked about their employment, work experience, education, health, family history, finances, attitudes and behaviors, and region of residence [20]. To create a detailed health profile of the cohort, the 40-and-over Health Module (“Health at 40”) was introduced in 1998 [21], and asked to participants in the survey year that they turned 40 years old. The module consisted of a depression symptom assessment; health professional consultation(s), the health status of participants’ parents; the 12-item Short Form Survey (SF-12); and a list of health conditions [21]. In 2008, a 50-and-over Health Module was introduced (“Health at 50”) with similar questions to the Health at 40 Module.

#### Depressive symptoms

Depressive symptoms were measured using an adapted version of the *Center for Epidemiologic Studies Depression Scale Short Form* (CES-D-SF), a self-reported scale used to measure depressive symptoms in the general population [22]. The 20-item CES-D was first included in 1992 survey (Supplemental Table 1.1) when the sample was between 28 and 35 years old. In the 1994 survey, a shortened 7-item scale (CES-D-SF) was included and then repeated in the Health at 40 (1998–2006) and Health at 50 modules (2008–2018). CES-D-SF scores from each of these measurements were used, including from the 1992 measurement that was based on the same seven items. CES-D-SF scores range between 0 and 21. A score of ≥8/21 on the CES-D-SF behaves similarly to the long form CES-D for measuring clinical levels of depressive symptoms (specificity = 0.97, sensitivity = 0.69) [23].

#### Monthly labor force status

Monthly labor force status was defined by the National Longitudinal Survey Program as the labor force status of the first week of each month of each year from 1979 to 2018. In the initial derived labor force status variable, participants were grouped into: no information; not working (unemployed vs. out of the labor force could not be determined); associated with an employer, but periods of work for the employer are missing; unemployed (if a respondent is not working and part of the time is spent looking for work or on layoff); out of the labor force; active military service; or employed [24]. Labor force states were further detailed and created by the study team based on participant death; if health status impacted the ability to work; the survey administrators reported a participant was not to be resurveyed; if a participant retired; and incarceration status (Table 1).

**Table 1:** Labor force status definition and sample sizes

State in multistate model	State	Definition	*N* monthly states, starting at age 30
Employed	Employed	Reported having a job	2,045,289
Unemployed	Unemployed	Reported that they were not working, as defined by the NLSY79 work history program (unemployed, and part of their time was spent looking for work), OR reported being on a layoff	107,195
Suboptimal labor force participation	Loosely attached to the labor force	Defined as associated with employer, OR	1576
		Reported having a job, but also considered themselves retired, OR	3922
		Not working (unemployment vs. out of the labor force unclear), OR	9448
		Not working, but also considered themselves retired, OR	28
		Unemployed, but also considered themselves retired	302
	Health status impacting ability to work	Reported that health status was impacting their ability to work, OR	37,841
		Reported that their health status was impacting their ability to work, but also considered themselves retired, OR	1770
		No labor force information was available, but reported that health status was impacting their ability to work	1442
Out of the Labor Force	Out of the labor force	Defined as out of the labor force by NLSY79 work history program; OR	464,552
		No labor force information was available, and participant was incarcerated, OR	221
		Out of the labor force, and participant was incarcerated, OR	9689
		Out of the labor force, reported retirement	9681
	Active military service	Associated with the military, as defined by NLSY79 work history program	10,723
Dead	Dead	Passed away, starting in 1993	818

#### Race/ethnicity and highest level of education

NLSY79 participants’ race/ethnicity was grouped into three categories: Hispanic (*n* = 1810) defined as Mexican American, Chicano, Mexican, Cuban, Puerto Rican, other Latin American/Spanish descent; Black (*n* = 2776); and non-Black/non-Hispanic (*n* = 4620), defined as White (~81%, *n* = 3756) or other descent (Japanese, Chinese, Vietnamese, Asian Indian, Native American, Korean, Inuit, Pacific Islander, or other races). Highest level of education at age 30 was grouped into 3 categories: less than high school; high school diploma; and college diploma (reported having completed ≥ 4 years of college).

#### Sociodemographic, health, and labor-related factors

Supplemental analyses explored factors that may influence depression and labor market experiences [25–28]. Baseline factors (1979) included participant birth year, gender, country of birth, state region, residing in a rural location, if their parents were still living, working status at age fourteen, family size, highest grade completed, total net family income, and family poverty status. We also explored markers of illegal activity and alcohol use. Sociodemographic factors measured over time (age 20, 30, 40, and 50), health factors (measured at age 40 and 50), and labor market indicators (measured from age 18 to 62) were also explored. Supplemental file 2 contains a detailed description of these variables.

### Analysis

Growth mixture modeling quantified depressive symptom trajectories [29, 30]. The 1992 CES-D-SF score was considered time T0_;_ the 1994 CES-D-SF score was time T+2; the Health at 40 CES-D-SF score was time T+9 (aligning to the midpoint year 2001, between 1997 and 2004 when NLSY79 participants turned 40 years old); and the Health at 50 CES-D-SF score was assessment time T+19 (aligning to the midpoint year of 2011, between 2007 and 2014 when NLSY79 participants turned 50 years old).

Unconditional growth models, specifying an intercept, linear, quadratic, and cubic slopes, with the variances of the growth factors fixed [31] and allowed to vary, were explored in R software (version 4.2.1) using the *lcmm* package [32]. CES-D-SF scores were modeled on a continuous scale, ranging from 0 to 21. For participants with one or more missing CES-D-SF scores, the missing score was imputed with the full information maximum likelihood estimator. To determine the most appropriate model, model fit criteria (i.e., lower AIC and BIC values and entropy values over 0.80 being favorable) and generalizability factors (i.e., alignment with clinical depression profiles, size of the smallest latent trajectory class, etc.) were considered.

Once the most appropriate model was selected, differences in baseline demographic and health factors by trajectory class were explored using Chi-square tests for categorical variables and ANOVA tests for continuous variables. Time-varying demographic and health factors are presented for age 20, 30, 40, and 50. Descriptive statistics for labor-related variables were calculated annually, starting around age 20.

**Fig. 1:**
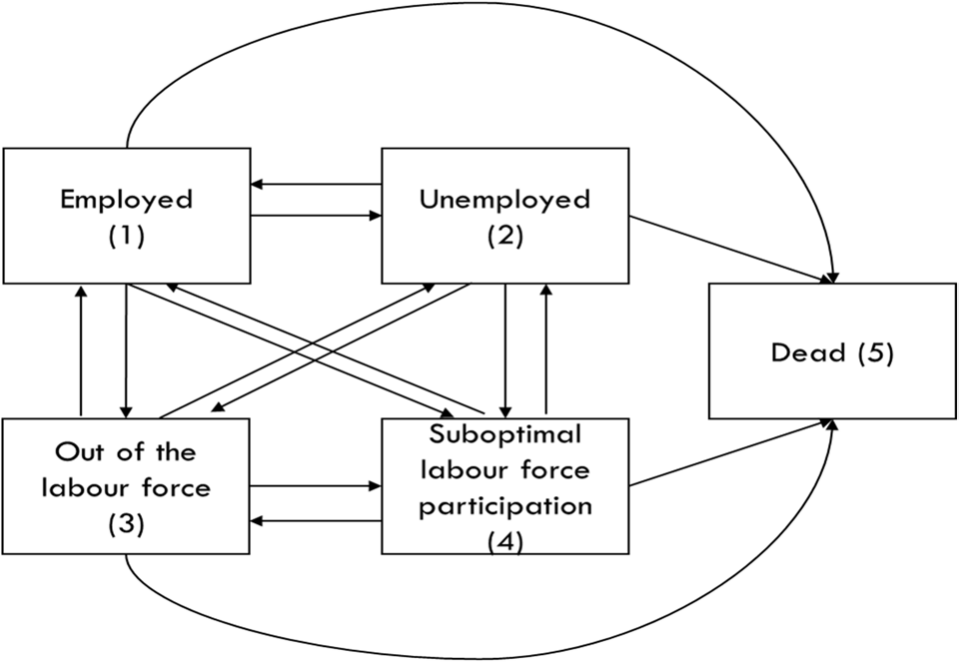
Multistate model

For estimating WLE, data were first stratified by gender and depressive symptom trajectory. In each group, a 5-state continuous, time non-homogenous Markov multistate model was fit using the van den Hout method [33]. Figure 1 highlights the multistate model. A participant could move in either direction between the four labor-related states. Death was an absorbing state, possible to access from all other states. Definitions of labor force states are listed in Table 1. As the follow-up of NLSY79 participants ended at age 62, we treated retirement as a component of being out of the labor force, rather than as an absorbing state.

From the multistate model, we estimated WLE using the *elect* R package [34] in 5-/10-year increments between the age of 30 and 60 using 500 simulations. We defined WLE as the number of years expected to remain in employment at a specific age, conditional on being in employment [34–36]. In WLE estimation, the maximum follow-up age was defined as 70 years old. Mean sojourn time in a labor force state was also predicted.

Two additional multistate models were explored: one to estimate WLE for each race/ethnicity and gender in each depressive symptom trajectory, and one to estimate WLE stratified by each educational level and gender in each trajectory. This was completed by adding race/ethnicity and education level as time-varying predictors in each multistate model.

## Results

### Latent depressive symptom trajectories

**Fig. 2:**
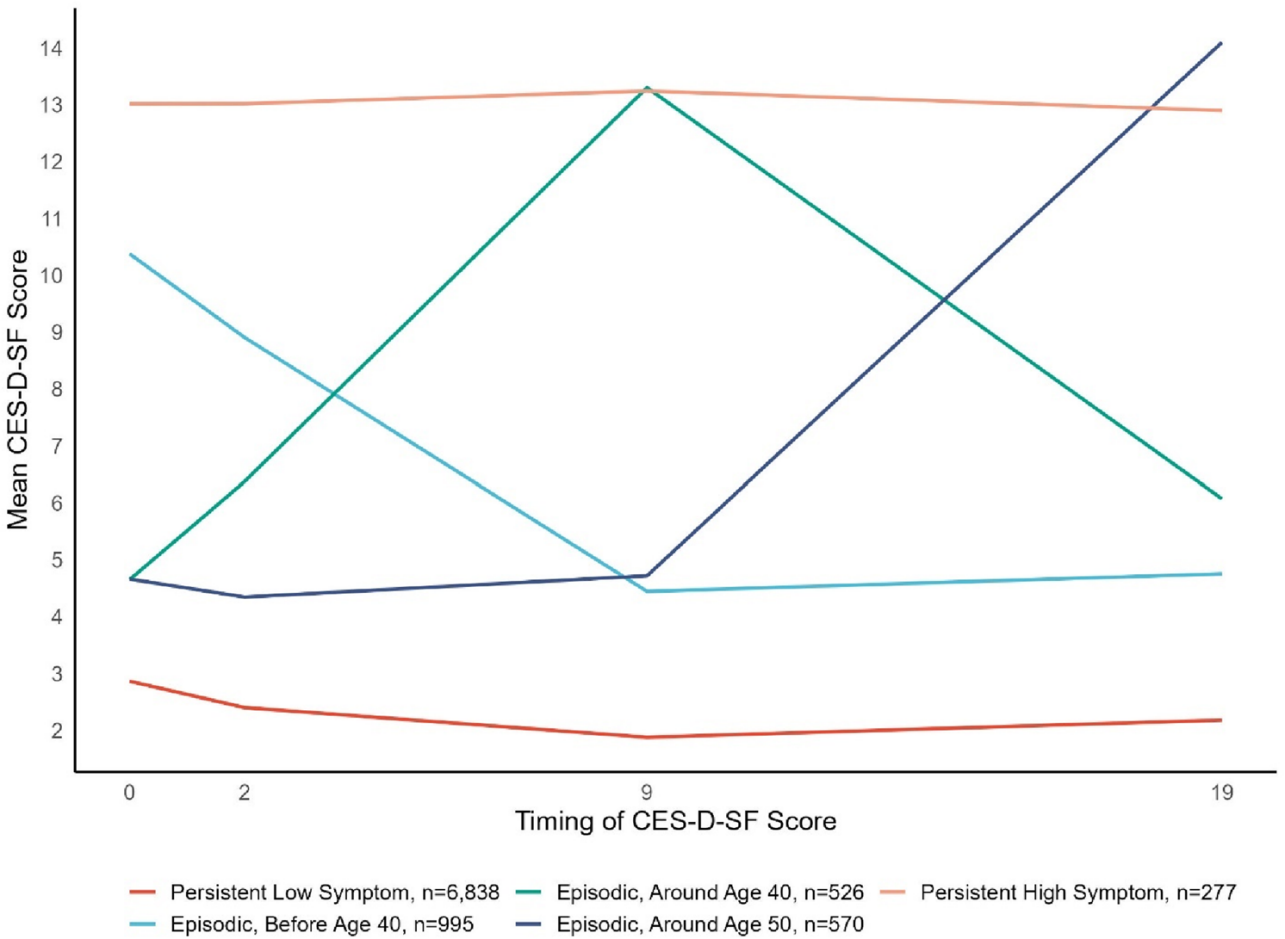
Average CES-D-SF scores, stratified by latent trajectory membership, NLSY79 sample (*n* = 9206)

Based on model fit statistics (AIC = 17,449, BIC = 179,592, entropy = 0.86, Supplemental Table 1.2), the size of latent classes, and alignment to clinical profiles and literature, the fixed-variance, 5-class quadratic slope model was deemed the most appropriate (Fig. 2). Classes 1 and 5 had relatively stable depression trajectories over time, while classes 2, 3, and 4 showed an episodic pattern of depression symptoms. Specifically, class 1, labeled persistent low depression symptoms, encompassed about 74% of the sample (*n* = 6838) and was characterized by low CES-D-SF scores (~ 2) at all time points. The remaining 26% of the sample had depression symptoms at some point in their lives, with most respondents (23%) reporting an episodic pattern. Class 2, (11% of the sample, *n* = 995), experienced an episodic depressive symptom trajectory, with high levels of depressive symptoms before age 40, followed by a decrease in depressive symptoms over the remaining assessments (labeled episodic, depression before age 40). Class 3 was labeled episodic, depression around age 40 (6%, *n* = 526) and class 4 was labeled episodic, depression around age 50 (6%, *n* = 570). Class 3 participants had low CES-D-SF scores until about age 40 years (T9, Health at 40 assessment), and Class 4 had low depressive symptoms until about age 50 when they had the highest CES-D-SF scores (T19, Health at 50 assessment). Class 5, (3%, *n* = 277) had stable and high, average ~ 13 CES-D-SF scores across the time period and was labeled persistent high depressive symptoms.

Supplemental File 2 highlights the sociodemographic, health, and labor market information for each latent trajectory class. Briefly, the persistent low symptom class was comprised of ~ 50% men, whereas the other classes were comprised of ~ 26–40% men. A lower proportion of Black or Hispanic participants were in the persistent low symptom class compared to the episodic or persistent high symptom trajectory classes. At age 30, while most participants in all classes reported completing a high school education, ~21% of those in the persistent low symptom class—versus ~ 8–9% in the episodic trajectories and ~ 4% in the persistent high symptom trajectory—had earned a college degree as their highest level of education. When exploring health status at age 40 and 50, a gradient effect was found with those in the low symptom trajectory having the best levels of health (e.g., an absence of chronic conditions and good physical and mental health SF-12 scores) [37], followed by the episodic trajectories, and then the persistent high depression symptom trajectory. This gradient effect was also present for labor market indicators (e.g., employment earnings, social insurance income, number of jobs and hours worked) from the early 20s to early 60s.

### Working life expectancy estimates

The 9,206 participants were followed for 214,378 person-years (2,572,534 person-months) from age 30 to the end of cohort follow-up (Table 2); 818 participants died over this period. Men (20.3 person-years) and women (18.9 person-years) in the persistent low symptom trajectory had the highest average person-years spent in employment, followed by the episodic before age 40 trajectory (men = 16.1 person-years, women = 16.8 person-years) and the episodic around age 40 and 50 trajectories. Those in the persistent high symptom trajectory spent the least amount of time in employment (men = 10.0 person-years, women = 10.4 person-years).

**Table 2:** Distribution of labor force states, by depressive symptom trajectory

	Sample *N*	Total person-moths	Total person-years	Average person-years				*N*, died	Difference in average employment years
				Employed	Unemployed	Out of the labor force	Health status impacting ability to work		
*n*	9206	2572534	214378	18.5	1.0	4.5	0.5	818	
Overall									
Persistent low symptom	6838	1986425	165535	19.6	0.8	3.4	0.3	517	Ref.
Episodic, before 40	995	294887	24574	16.5	1.3	6.0	0.8	120	− 3.1
Episodic, age 40	526	158690	13224	14.9	1.4	7.9	0.9	81	− 4.8
Episodic, age 50	570	182791	15233	16.0	1.4	8.3	1.0	44	− 3.6
Persistent high symptom	277	81704	6809	10.3	1.4	11.0	1.9	56	− 9.3
Men									
Persistent low symptom	3635	1041367	86781	20.3	0.9	2.4	0.3	328	Ref.
Episodic, before 40	380	108118	9010	16.1	1.7	5.0	0.9	54	− 4.2
Episodic, age 40	212	61243	5104	14.9	1.5	6.7	1.0	41	− 5.4
Episodic, age 50	217	67590	5633	15.8	1.5	7.7	1.0	24	− 4.5
Persistent high symptom	73	20594	1716	10.0	1.8	9.6	2.1	17	− 10.3
Women									
Persistent low symptom	3203	945058	78755	18.9	0.7	4.6	0.3	189	Ref.
Episodic, before 40	615	186769	15564	16.8	1.1	6.6	0.8	66	− 2.1
Episodic, age 40	314	97447	8121	14.8	1.4	8.7	0.9	40	− 4.1
Episodic, age 50	353	115201	9600	16.2	1.3	8.7	1.0	20	− 2.8
Persistent high symptom	204	61110	5093	10.4	1.3	11.5	1.8	39	− 8.5
Men: Black									
Persistent low symptom	1043	302799	25233	18.7	1.5	3.6	0.5	117	Ref.
Episodic, before 40	150	43072	3589	13.1	2.4	7.3	1.1	28	− 5.5
Episodic, age 40	83	24143	2012	13.1	1.7	8.3	1.2	24	− 5.5
Episodic, age 50	65	20276	1690	14.3	2.3	8.6	0.8	9	− 4.4
Persistent high symptom	22	6619	552	7.5	2.6	12.3	2.7	3	− 11.1
Men: Hispanic									
Persistent low symptom	697	198431	16536	19.7	1.1	2.5	0.4	67	Ref.
Episodic, before 40	73	20592	1716	17.5	1.5	3.6	0.8	9	– 2.2
Episodic, Age 40	44	12759	1063	13.3	1.4	8.3	1.2	3	– 6.4
Episodic, Age 50	49	15474	1290	15.8	1.5	7.9	1.0	5	– 3.9
Persistent high symptom	20	5822	485	10.3	1.9	10.1	1.9	5	– 9.4
Men: White									
Persistent low symptom	1895	540137	45011	21.3	0.6	1.6	0.2	144	Ref.
Episodic, before 40	157	44454	3705	18.2	1.2	3.6	0.7	17	– 3.1
Episodic, age 40	85	24341	2028	17.4	1.4	4.3	0.8	14	– 4.0
Episodic, age 50	103	31840	2653	16.7	1.0	6.9	1.1	10	– 4.6
Persistent high symptom	31	8153	679	11.4	1.3	7.3	1.9	9	– 9.9
Women: Black									
Persistent low symptom	893	270724	22560	19.1	1.2	4.5	0.5	75	Ref.
Episodic, before 40	211	65564	5464	16.9	1.5	6.5	1.0	27	– 2.2
Episodic, age 40	121	38186	3182	14.3	1.8	9.4	0.8	16	– 4.8
Episodic, age 50	120	38847	3237	14.6	1.6	9.8	1.0	8	– 4.5
Persistent high symptom	68	20930	1744	10.2	1.6	11.7	2.1	11	– 8.9
Women: Hispanic									
Persistent low symptom	641	186104	15509	18.1	0.7	5.0	0.3	33	Ref.
Episodic, before 40	125	37779	3148	16.5	1.0	7.1	0.6	9	– 1.6
Episodic, age 40	53	16648	1387	15.2	1.5	8.4	1.1	5	– 3.0
Episodic, age 50	56	18149	1512	13.8	1.4	10.6	1.2	2	– 4.4
Persistent high symptom	52	15466	1289	8.4	1.1	13.5	1.8	8	– 9.8
Women: White									
Persistent low symptom	1669	488230	40686	19.1	0.5	4.5	0.3	81	Ref.
Episodic, before 40	279	83426	6952	16.9	0.9	6.4	0.7	30	– 2.2
Episodic, age 40	140	42613	3551	15.2	1.1	8.3	0.8	19	– 3.9
Episodic, age 50	177	58205	4850	18.0	1.1	7.5	0.9	10	– 1.1
Persistent high symptom	84	24714	2060	11.8	1.2	10.0	1.6	20	– 7.3
Men: less than high school									
Persistent low symptom	499	139456	11621	17.5	1.6	3.7	0.5	71	Ref.
Episodic, before 40	86	23882	1990	12.4	2.2	7.5	1.1	20	– 5.1
Episodic, age 40	68	19902	1659	11.1	1.6	10.1	1.5	12	– 6.4
Episodic, age 50	62	18906	1576	12.4	2.1	9.7	1.3	7	– 5.1
Persistent high symptom	31	9328	777	10.7	2.1	9.9	2.4	7	– 6.8
Men: high school diploma									
Persistent low symptom	2202	643117	53593	20.5	1.0	2.5	0.3	196	Ref.
Episodic, before 40	237	69673	5806	17.3	1.6	4.6	0.9	29	– 3.2
Episodic, age 40	122	34546	2879	15.8	1.5	5.4	0.8	26	– 4.7
Episodic, age 50	127	41068	3422	17.6	1.4	6.9	0.9	15	– 2.9
Persistent high symptom	33	9482	790	9.1	1.7	11.0	2.2	8	– 11.5
Men: college degree									
Persistent low symptom	728	210084	17507	25.5	0.4	1.0	0.2	33	Ref.
Episodic, before 40	24	7361	613	22.7	0.8	1.7	0.4	2	0.1
Episodic, age 40	12	3927	327	24.5	0.9	1.8	0.1	1	2.0
Episodic, age 50	13	4417	368	22.2	1.4	4.4	0.4	0	– 0.4
Persistent high symptom	3	1019	85	27.1	1.0	0.1	0.0	0	4.6
Women: less than high school									
Persistent low symptom	318	93297	7775	14.3	1.3	8.2	0.7	43	Ref.
Episodic, before 40	112	32941	2745	11.7	1.2	10.3	1.3	17	– 2.6
Episodic, age 40	61	19505	1625	10.6	1.6	12.9	1.4	10	– 3.6
Episodic, age 50	55	16945	1412	11.0	1.4	12.2	1.2	2	– 3.3
Persistent high symptom	66	20474	1706	7.2	1.1	15.3	2.2	14	– 7.1
Women: high school diploma									
Persistent low symptom	2044	615253	51271	19.5	0.8	4.5	0.4	110	Ref.
Episodic, before 40	420	126162	10514	17.5	1.1	5.7	0.7	42	– 2.0
Episodic, age 40	208	63855	5321	15.3	1.4	8.0	0.8	26	– 4.2
Episodic, age 50	245	81261	6772	16.8	1.5	8.4	1.0	15	– 2.8
Persistent high symptom	124	37240	3103	12.3	1.3	9.8	1.7	20	– 7.2
Women: college degree									
Persistent low symptom	684	199355	16613	20.3	0.3	3.5	0.2	25	Ref.
Episodic, before 40	65	20013	1668	20.0	0.8	4.3	0.5	3	– 0.3
Episodic, age 40	30	9633	803	20.7	0.7	5.0	0.4	2	0.4
Episodic, age 50	36	12219	1018	21.2	0.7	5.5	0.8	3	0.9
Persistent high symptom	9	2148	179	10.0	1.6	7.0	1.3	3	– 10.4

*Ref* reference

**Fig. 3:**
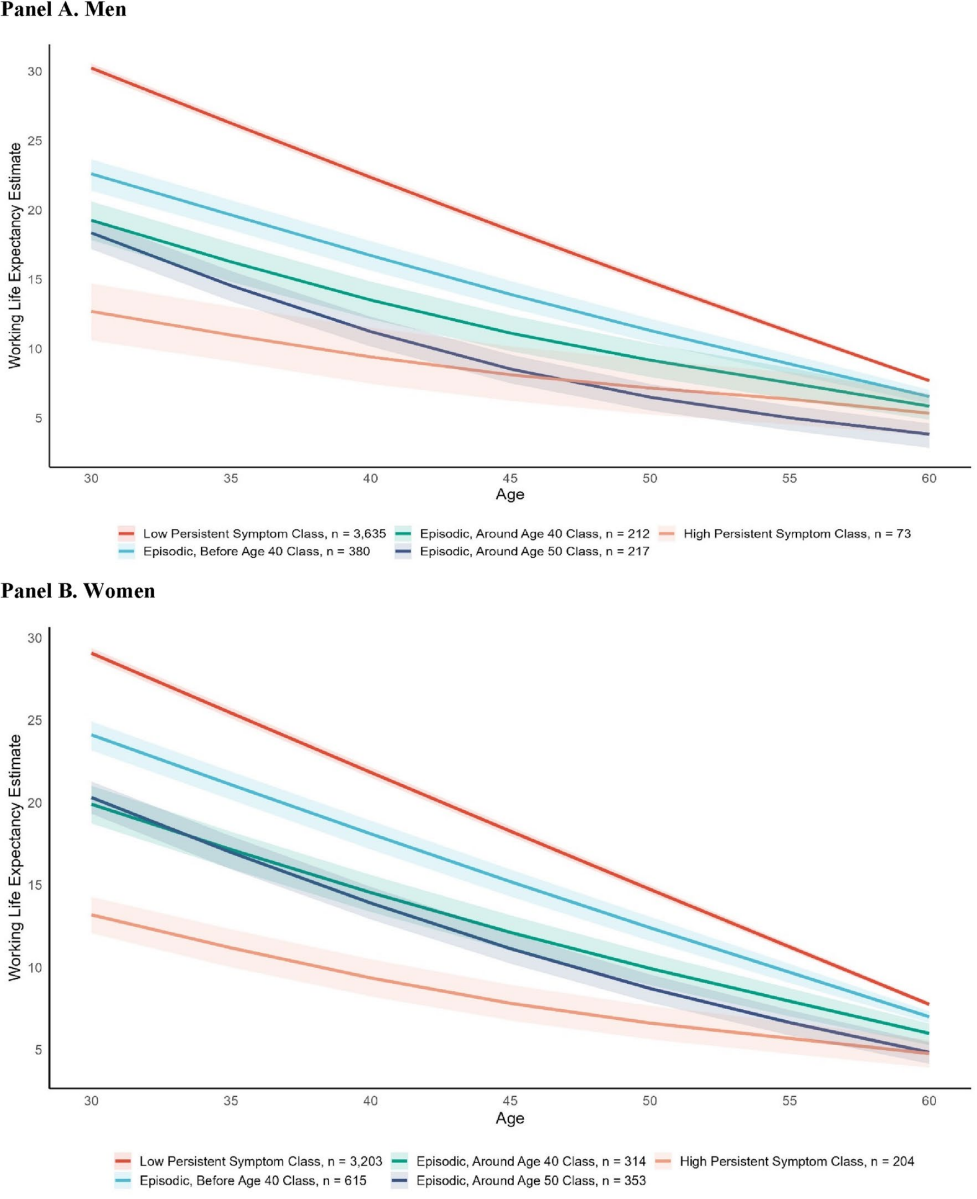
Estimated working life expectancy by gender and depressive symptom trajectory class

For all trajectory classes, WLE were highest at age 30 and decreased until age 60, and gender differences within trajectory classes were not present (Fig. 3, Supplemental Table 1.3). However, differences in WLE estimates were present between age 30 and 55 within symptom trajectories, and between depressive symptom trajectories. For both men and women, the persistent low symptom trajectory class had the highest WLE between age 30 and 60. For example, the WLE for men in the low symptom trajectory was estimated to be 30.3 years at age 30; in other words, these men could be expected to work another 30.3 years given they were already in employment at age 30. The next highest WLE was seen in the episodic before age 40 trajectory class, followed by the episodic around age 40 and 50 trajectories. The persistent high symptom class had the lowest WLE. For men in the persistent high symptom class, the WLE was estimated to be 13.2 years at age 30, 9.7 years at age 40, 7.5 years at age 50, and 5.7 years at age 60. Sojourn time estimates showed that participants in the persistent low symptom trajectory spent a longer amount of time in employment before moving to another state (men ~ 7.2 years, women ~ 5.7 years, Supplemental Table 1.4) compared to those in the episodic trajectories (~ 4 years for men and women) and persistent high symptom trajectory (men~3.1 years, women~2.6 years).

A detailed description of WLE estimates stratified by race/ethnicity, education, gender, and depression trajectory are presented in Supplemental File 1. The trend that men and women in the low symptom trajectory had the highest WLE and those in the persistent high symptom trajectory had the lowest WLE generally remained. However, WLE for Black men in the episodic before age 40 (WLE_age 30_ = 17.5), around age 40 (WLE_age 30_ = 15.5), and around age 50 (WLE_age 30_ = 15.6) were all lower than NBNH participants in the low symptom trajectory (WLE=32.6) or the episodic, before 40 trajectory (WLE = 26.5). For Hispanic men, the WLE at age 30 of those in the episodic, before age 40 trajectory (WLE = 24.3) was similar to WLEs for NBNH participants in the same trajectory. Among women, WLE for Black, Hispanic, and NBNH women were similar for those in the low symptom and episodic, before age 40 trajectories. In the episodic, age 50 trajectory, Black (WLE = 18.2) and Hispanic (WLE = 16.0) women had lower WLE at age 30 compared to NBNH (WLE = 22.3). For participants with less than a high school education, at age 30 there was a ~ 8-year difference in WLE between those in the persistent low symptom and episodic, before age 40 trajectory among men (difference among women was ~ 4 years). This difference was ~6 years for men with a high school diploma and ~4 years for women.

## Discussion

This study is among the first to estimate the WLE specifically examining various courses of depression by gender, race/ethnicity, and education level. The modeling of monthly labor force status beginning at age 30 until the late 50s/early 60s is a clear strength of this study, allowing for a robust, nuanced labor profile of this cohort. The findings of this study indicated that ~ 26% of individuals in the working-age population experienced high levels of depressive symptoms at some point between the late 20s and early 50s, with ~ 3% experiencing persistent, high depressive symptoms over 20 years. However, despite the fact that individuals in the episodic trajectories experienced periods of mental well-being, they still experienced losses to WLE, particularly when episodes occurred around age 40 or 50. Although the lifetime prevalence of high levels of depressive symptoms was higher in women relative to men (31.7% vs. 19.5%), the study findings also indicated that, generally, women and men were similar in their WLE estimates. Race/ethnicity and education were shown to be important factors that impacted WLE among those with depression, particularly among Black men who experienced any depression trajectory course, and for those with less than a high school education.

Results indicated that most adults who experienced depression in the cohort between the late 20s and early 50s did not have persistent symptoms, but experienced depression on an episodic basis, aligning to prior literature [38]. These findings are important and highlight the challenges people living with even occasional bouts of depression face in retaining employment through the prime working years. They also point to areas where employers, labor organizations, policy makers, and health professionals should focus to consider labor practices and policies to improve clinical and vocational support to adults experiencing depression, enforce workplace practices to reduce the risk of symptoms attributable to work exposures, and increase access to health care and psychological therapy to minimize the severity of depressive episodes.

This study enhances prior literature by including different episodic trajectories, which indicate a smaller difference in WLE when compared to non-depressed working-aged adults in prior literature. Plana-Ripoll and colleague reported that Danish individuals with a diagnosed single or recurrent depressive disorder had 10.34 total working years lost [39], which is similar to the difference in average employment years between the persistent high and persistent low depressive trajectory groups in our study (− 9.3 years). When depressive episodes are in remission, individuals may function within the labor force in ways that favor steady and attached employment [40], leading to the smaller difference in WLE. For example, Luo and colleagues found that individuals with a persistent remission of depressive symptoms (defined as their last episode was 2–3 years prior) showed labor force participation similar to those with no history of depression [3].

Participants in the episodic depression trajectories spent a greater proportion of time out of the labor force than the low symptom trajectory participants, and they spent shorter durations of time employed. This highlights not only the age at which a depressive episode may occur but also the importance of job transitions and turnover. Research on disability and employment highlights that many individuals living with chronic physical and mental health conditions experience job insecurity and transitions in work [41, 42]. Under the Americans with Disabilities Act & Amendments Act, depression qualifies as a mental disability [43]. Although legislation identifies people living with mental health conditions as a group protected against discrimination and is intended to help individuals with depression remain employed, the frequent transitions in jobs and shorter durations of time employed that we uncovered in this study may suggest that workplace practices and supports may be lagging for people with depression. Additional attention and research on this issue is necessary, particularly understanding workplace accommodation processes for disability claims [44].

The trajectory with high symptoms before age 40 had a more favorable WLE than the other two episodic trajectories. Health shocks, such as experiencing a depressive episode, during the sixth decade of life may influence premature labor market exit or retirement [6, 45]. These older workers may have increased eligibility for labor market exit (e.g., early retirement eligibility or employer pension options), reducing the severity of the labor force exit compared to an exit in younger adulthood [46]. Therefore, workplace and public mental health interventions may want to focus on the unique stressors of this age group to reduce the likelihood of depressive episodes, or severity if they occur.

Individuals with the most severe course of depressive symptoms had the lowest WLE. Despite the small percentage of individuals in this group, the impact of depression on their lives is the most profound. In a network meta-analysis exploring interventions to obtain and maintain employment among the working-aged population with severe mental illness, supported employment programs (programs and services that help individuals receive and maintain employment) [47, 48] were found to be the most effective interventions to gaining competitive employment [49]. Unfortunately, these programs are not widely available, and individuals may have difficulty accessing them.

Our study highlights clear racialized and education-related differences that persist in WLE, aligning with previous literature [15, 16, 50–53]. These differences not only impact labor market experience in adults without mental health challenges [52, 54, 55], but they play a heightened role in reducing working years and other labor market outcomes [56] among those with depression symptoms. The trends seen within and between education groups supports the cumulative advantage/disadvantage theory: [57] experiencing a lower educational attainment in emerging adulthood may not only increase the likelihood of unemployment or experiencing high depressive symptoms at some point during the prime working years [5, 58], but also increase the likelihood of that a depressive episode may immediately impact job stability and other labor market outcomes [15]. Conversely, gaining a higher level of education prior to starting one’s career may act as a buffer from subsequent depressive episodes, potentially through having more job security surrounding a depressive episode.

Over the past 30 years, stratification economic scholars have posited that racial discrimination preserves social hierarchies, leading to long-standing labor inequalities [59]. These hierarchies are maintained at a structural level through processes such as group formation, group identity, and group action [60, 61]. Through this lens, one pathway that may be influencing the presented findings is through healthcare access and behaviors. For example, members of Hispanic or Black communities often access mental health services later, or less frequently than White communities [62, 63] due to stigma, fear of health or other social ramifications, and scarce amount of culturally appropriate mental health professionals and treatment options available [63–65]. As such, individuals from these communities may spend longer amounts of time in depressive states, potentially impacting their health capital and ability to work. This suggests the need to focus not only on workers with depression and getting them treatment and resources to be successful in their educational attainment and health, but also the need to address systemic barriers that impact significant numbers of Americans.

### Generalizability of findings

The findings of this study are based on over 30 years of life experience, measured in cohort of individuals born before 1965. This raises the question: will these findings be similar in more recent generations of Americans? The best, and most accurate, answer will come from replicating this study in more recent cohorts. However, we briefly highlight some context of the sample, American labor trends, and depression prevalence trends.

The NLSY79 participants entered their prime working years (~ 30 years old) between 1988 and 1995, where their educational and employment choices were potentially influenced by the 1981–1982 and 1990–1991 American recessions. The recession of the early 1980s was the most severe since the Great recession [66], and the recession of the early 1990s was the first time that a decline in white collar jobs, and employment held by women was seen [67]. Labor trends that will more greatly impact younger cohorts include the 2007–2009 recession and wage stagnation [68], which have the potential to increase disparities in WLE between those with and without depression. Related, at age 30, roughly ~ 60% of NLSY79 participants had a high school diploma. In 2001, roughly 94% of Americans aged 25–29 had at least a high school diploma, with ~ 39% having at least an undergraduate college degree [69]. The increase in educational attainment of the population has the potential to increase WLE [70] among all trajectories seen in this study; however, discrepancies between episodic and persistent high symptom trajectories may likely remain [56].

Literature exploring trends in depression prevalence have shown that depression prevalence in the general population remained stable until the early 2000s [71–73], however has potentially increased since [74–76]. Cohort effects have also been seen, with younger cohorts having higher depression prevalence earlier in life. From the 1990s until 2015, significant increase in treatment access and options were seen, which have since remained stable [76]. If younger cohorts have earlier, accessible, and effect treatment options, this may reduce disparities in WLE. However, if accessible treatment is not available, this has the potential to increase the number and severity of depressive episodes over time, potentially widening disparities in WLE. Lastly, it is important to recognize that racialized groups and other vulnerable groups are more likely to displaced from the labor force [77] and have reduced access to treatment [78], which has the potential to increase WLE disparities.

### Limitations

Despite the strengths of this research, limitations include that the CES-D-SF is not a clinical instrument for major depressive episodes. Participant answers could have been influenced by social desirability bias, stigma, or the presence of other chronic conditions. If this bias is present, the size of trajectory groups could be underestimated. Second, some of the missingness in the sample may not be random, as men (particularly those who were not Black or not Hispanic) and older cohort members in 1979 were more likely to be excluded. If this bias is present, it may have led to an overestimation of the number of depressive symptom trajectories (as the models were unconditional), size of trajectories, and magnitude of WLE estimates.

Despite having up to 30 years of monthly labor force follow-up in the multistate models, the overall sample sizes used to estimate WLE stratified by race/ethnicity or education level, gender, and depressive symptoms were relatively small, particularly for the persistent high symptom trajectory, which may impact the internal and external validity of our findings. This precluded the modeling of covariates, and having to condense the college degree and high school diploma groups for WLE estimates of varying educational level among men in the persistent high symptom trajectory. In addition, the distribution of covariates within each trajectory may differ, resulting in inflated WLE estimates.

Another limitation of this study and others that use multistate modeling is that researchers must make numerous assumptions (e.g., the number of possible labor force states, beginning multistate models at age 30, assuming multistate models follow a Markov assumption where the future evolution of one’s labor force trajectory is dependent on one’s current labor force state). Many of these assumptions are used in other studies; e.g., [36, 79–81] further research is needed to understand the potential bias these assumptions place within WLE.

## Conclusions

The present study uncovers how individuals experiencing various courses of depression participate in the labor force during the prime working years. Findings highlight the heterogeneity of labor force experiences for men and women with episodic and persistent depression compared to no depression throughout adulthood. This study provides evidence that can be used as a benchmark to assist health economists and labor policy makers in improving employment opportunities and sustainability among ~ 21.9 million American adults who experience depression [1]. Findings have the potential to inform labor and mental health initiatives, including health equity legislation and other systemic barriers, to help prevent and mitigate impacts of depressive episodes. Results also point to examining how the onset age and trajectory of depression may impact labor market participation and supports differently over time.

### Supplementary Information

Below is the link to the electronic supplementary material.

Supplementary file1 (DOCX 886 KB)

Supplementary file2 (DOCX 762 KB)

## Data Availability

Information and data from the National Longitudinal Surveys Program can be found at https://www.nlsinfo.org/.
