# Supplementary material for: The working life expectancy of American adults experiencing depression
Source: Soc Psychiatry Psychiatr Epidemiol. 2023 Sep 7;59(6):1013–27. doi: 10.1007/s00127-023-02547-4 (PMC11116182; doi:10.1007/s00127-023-02547-4)
Supplement: Supplementary file 1 — Supplementary file1 (DOCX 886 KB) [file 127_2023_2547_MOESM1_ESM.docx]

**The Working Life Expectancy of American Adults Experiencing Depression**

**Supplemental File 1**

**July 2023**

**Supplemental Figure 1.1 Study Inclusion Criteria**

**Supplemental Table 1.1. CES-D Questions in the NLSY79, by Survey Year/Survey Module**

| # | Question/Prompt: | Survey Year/Module | | | |
| --- | --- | --- | --- | --- | --- |
|  | *I am going to read a list of the ways you might have felt or behaved* | **1992** | **1994** | **Health at 40 (1997-2004)** | **Health at 50 (2007-2014)** |
|  | *recently. After each statement, please tell me how often you felt this way during the past week.* |  |  |  |  |
| 1 | I was bothered by things that usually don't bother me. | x |  |  |  |
| 2 | **I did not feel like eating; my appetite was poor. (Somatic)** | **x** | **x** | **x** | **x** |
| 3 | I felt that I couldn't shake off the blues even with help from my family and friends. | x |  | x | x |
| 4 | I felt that I was just as good as other people. | x |  |  |  |
| 5 | **I had trouble keeping my mind on what I was doing. (Somatic)** | **x** | **x** | **x** | **x** |
| 6 | **I felt depressed. (Negative affect)** | **x** | **x** | **x** | **x** |
| 7 | **I felt that everything I did was an effort. (Somatic)** | **x** | **x** | **x** | **x** |
| 8 | I felt hopeful about the future. | x |  |  |  |
| 9 | I thought my life had been a failure. | x |  |  |  |
| 10 | I felt fearful. | x |  |  |  |
| 11 | **My sleep was restless. (Somatic)** | **x** | **x** | **x** | **x** |
| 12 | I was happy. | x |  |  |  |
| 13 | I talked less than usual. | x |  |  |  |
| 14 | I felt lonely. | x |  | x | x |
| 15 | People were unfriendly. | x |  |  |  |
| 16 | I enjoyed life. | x |  |  |  |
| 17 | I had crying spells. | x |  |  |  |
| 18 | **I felt sad. (Negative affect)** | **x** | **x** | **x** | **x** |
| 19 | I felt that people dislike me. | x |  |  |  |
| 20 | **I could not get "going." (Somatic)** | **x** | **x** | **x** | **x** |

***Bold “X” denotes questions used in CES-D-SF Score***

**Supplemental Table 1.2. Latent Class Growth Trajectory Modeling, Model Fit Statistics**

| Model | Model Fit | | | | |  |  | Class Size (%) (n=9,206) | | | | | | |
| --- | --- | --- | --- | --- | --- | --- | --- | --- | --- | --- | --- | --- | --- | --- |
| N, Classes | log-likelihood | N, Parameters | AIC | BIC | entropy | LMR-LRT | LMR-LRT, p | Class 1 | Class 2 | Class 3 | Class 4 | Class 5 | Class 6 | Class 7 |
| 1 | -95089 | 4 | 190186 | 190214 | 1 | NA | NA | 100 | NA | NA | NA | NA | NA | NA |
| 2 | -91936 | 8 | 183888 | 183945 | 0.85 | 6083.3 | <0.001 | 84.14 | 15.86 | NA | NA | NA | NA | NA |
| 3 | -91085 | 12 | 182195 | 182280 | 0.86 | 1641.9 | <0.001 | 81.21 | 12.07 | 6.72 | NA | NA | NA | NA |
| 4 | -90341 | 16 | 180714 | 180828 | 0.86 | 1435.9 | <0.001 | 77.65 | 10.34 | 6.02 | 6.00 | NA | NA | NA |
| 5 | -89705 | 20 | 179449 | 179592 | 0.86 | 1228.2 | <0.001 | 74.28 | 10.81 | 6.19 | 5.71 | 3.01 | NA | NA |
| 6 | -89469 | 24 | 178985 | 179156 | 0.86 | 455.4 | <0.001 | 72.90 | 10.40 | 6.24 | 6.15 | 2.80 | 1.52 | NA |
| 7 | -89469 | 28 | 178993 | 179193 | 0.77 | 0.0 | 1.0 | 72.60 | 10.54 | 6.29 | 6.25 | 2.80 | 1.52 | 0.00 |

*Note: All models included a quadratic trajectory slope, and fixed variances of parameter estimates.*

**Supplemental Table 1.3 Working Life Expectancy Between Ages 30 to 60, Stratified by Gender, and Depressive Symptom Trajectory**

|  |  | Men | | |  | Women | | |
| --- | --- | --- | --- | --- | --- | --- | --- | --- |
| Trajectory Class | Age | WLE | 95% CI | |  | WLE | 95% CI | |
| Persistent Low Symptoms | 30 | 30.28 | 29.88 | 30.57 |  | 29.12 | 28.76 | 29.39 |
|  | 35 | 26.30 | 25.95 | 26.59 |  | 25.48 | 25.12 | 25.76 |
|  | 40 | 22.39 | 22.05 | 22.67 |  | 21.87 | 21.53 | 22.15 |
|  | 45 | 18.57 | 18.25 | 18.84 |  | 18.30 | 17.98 | 18.57 |
|  | 50 | 14.85 | 14.56 | 15.10 |  | 14.77 | 14.48 | 15.00 |
|  | 55 | 11.23 | 11.01 | 11.44 |  | 11.27 | 11.05 | 11.46 |
|  | 60 | 7.71 | 7.55 | 7.84 |  | 7.78 | 7.62 | 7.91 |
| Episodic, Before Age 40 | 30 | 22.84 | 21.40 | 23.65 |  | 24.31 | 23.18 | 24.93 |
|  | 35 | 19.82 | 18.57 | 20.71 |  | 21.25 | 20.19 | 21.89 |
|  | 40 | 16.87 | 15.64 | 17.76 |  | 18.25 | 17.17 | 18.91 |
|  | 45 | 14.05 | 12.94 | 14.89 |  | 15.34 | 14.31 | 15.95 |
|  | 50 | 11.43 | 10.46 | 12.16 |  | 12.53 | 11.61 | 13.07 |
|  | 55 | 9.00 | 8.19 | 9.59 |  | 9.80 | 9.06 | 10.22 |
|  | 60 | 6.61 | 6.06 | 7.03 |  | 7.07 | 6.63 | 7.34 |
| Episodic, Around Age 40 | 30 | 19.56 | 17.84 | 20.61 |  | 20.18 | 18.75 | 21.03 |
|  | 35 | 16.50 | 14.83 | 17.65 |  | 17.38 | 15.99 | 18.24 |
|  | 40 | 13.71 | 12.14 | 14.84 |  | 14.74 | 13.38 | 15.62 |
|  | 45 | 11.30 | 9.82 | 12.41 |  | 12.31 | 11.04 | 13.17 |
|  | 50 | 9.35 | 7.96 | 10.38 |  | 10.11 | 8.93 | 10.87 |
|  | 55 | 7.71 | 6.38 | 8.62 |  | 8.11 | 7.04 | 8.74 |
|  | 60 | 6.02 | 4.87 | 6.67 |  | 6.14 | 5.30 | 6.60 |
| Episodic, Around Age 50 | 30 | 18.55 | 17.19 | 19.38 |  | 20.57 | 19.34 | 21.29 |
|  | 35 | 14.70 | 13.42 | 15.61 |  | 17.19 | 15.96 | 17.97 |
|  | 40 | 11.35 | 10.16 | 12.31 |  | 14.09 | 12.92 | 14.90 |
|  | 45 | 8.63 | 7.51 | 9.58 |  | 11.31 | 10.24 | 12.08 |
|  | 50 | 6.59 | 5.54 | 7.45 |  | 8.88 | 7.88 | 9.58 |
|  | 55 | 5.11 | 4.10 | 5.89 |  | 6.81 | 5.88 | 7.42 |
|  | 60 | 3.96 | 2.84 | 4.61 |  | 4.99 | 4.16 | 5.48 |
| Persistent High Symptoms | 30 | 13.24 | 10.60 | 14.70 |  | 13.42 | 12.09 | 14.28 |
|  | 35 | 11.36 | 9.09 | 13.03 |  | 11.37 | 10.02 | 12.32 |
|  | 40 | 9.72 | 7.47 | 11.53 |  | 9.52 | 8.25 | 10.50 |
|  | 45 | 8.41 | 6.25 | 10.17 |  | 7.97 | 6.79 | 8.94 |
|  | 50 | 7.47 | 5.25 | 9.17 |  | 6.77 | 5.65 | 7.68 |
|  | 55 | 6.70 | 4.54 | 8.30 |  | 5.86 | 4.77 | 6.69 |
|  | 60 | 5.66 | 3.76 | 6.79 |  | 4.94 | 3.93 | 5.59 |

**Supplemental Table 1.4. Labor Force State Sojourn Time, Stratified by Gender, and Depressive Symptom Trajectory**

|  |  | Men | | |  | | Women | | | | |  |
| --- | --- | --- | --- | --- | --- | --- | --- | --- | --- | --- | --- | --- |
|  | Labor Force State | Est | 95% CI | |  | | Est | | 95%CI | | |  |
| Persistent Low Symptom | Employed | 7.22 | 7.08 | 7.36 | |  | | 5.72 | | 5.61 | 5.83 | |
|  | Unemployed | 0.58 | 0.56 | 0.59 | |  | | 0.51 | | 0.50 | 0.53 | |
|  | Out of the Labour Force | 1.21 | 1.18 | 1.24 | |  | | 1.53 | | 1.50 | 1.56 | |
|  | Suboptimal | 0.99 | 0.94 | 1.05 | |  | | 0.89 | | 0.84 | 0.94 | |
| Episodic, Before Age 40 | Employed | 4.12 | 3.91 | 4.33 | |  | | 4.04 | | 3.88 | 4.20 | |
|  | Unemployed | 0.67 | 0.63 | 0.72 | |  | | 0.53 | | 0.50 | 0.56 | |
|  | Out of the Labour Force | 1.60 | 1.51 | 1.69 | |  | | 1.65 | | 1.59 | 1.72 | |
|  | Suboptimal | 1.15 | 1.03 | 1.28 | |  | | 1.07 | | 0.98 | 1.18 | |
| Episodic, Age 40 | Employed | 4.15 | 3.86 | 4.47 | |  | | 3.56 | | 3.37 | 3.76 | |
|  | Unemployed | 0.62 | 0.57 | 0.67 | |  | | 0.56 | | 0.52 | 0.60 | |
|  | Out of the Labour Force | 2.19 | 2.02 | 2.38 | |  | | 2.06 | | 1.95 | 2.18 | |
|  | Suboptimal | 1.15 | 0.99 | 1.34 | |  | | 0.99 | | 0.88 | 1.12 | |
| Episodic, Age 50 | Employed | 4.07 | 3.79 | 4.36 | |  | | 3.79 | | 3.60 | 3.99 | |
|  | Unemployed | 0.67 | 0.61 | 0.73 | |  | | 0.61 | | 0.57 | 0.66 | |
|  | Out of the Labour Force | 2.21 | 2.04 | 2.39 | |  | | 2.06 | | 1.95 | 2.17 | |
|  | Suboptimal | 1.07 | 0.93 | 1.23 | |  | | 1.02 | | 0.92 | 1.14 | |
| Persistent High Symptoms | Employed | 3.11 | 2.72 | 3.56 | |  | | 2.56 | | 2.39 | 2.75 | |
|  | Unemployed | 0.83 | 0.71 | 0.97 | |  | | 0.54 | | 0.50 | 0.60 | |
|  | Out of the Labour Force | 2.62 | 2.31 | 2.97 | |  | | 2.57 | | 2.40 | 2.76 | |
|  | Suboptimal | 1.32 | 1.08 | 1.61 | |  | | 1.01 | | 0.90 | 1.13 | |

Est = Estimate
CI= Confidence Interval

**Supplemental Table 1.5. Working Life Expectancy Between Ages 30 to 60, Stratified by Gender, Race, and Depressive Symptom Trajectory**

|  |  |  | Men | | |  | Women | | |
| --- | --- | --- | --- | --- | --- | --- | --- | --- | --- |
| Trajectory Class | Race/Ethnicity | Age | WLE | 95%CI | |  | WLE | 95%CI | |
| Persistent Low Symptom | Black  (men: n=1,043, women: n=893) | 30 | 26.84 | 26.23 | 27.41 |  | 28.13 | 27.53 | 28.69 |
|  |  | 40 | 19.73 | 19.29 | 20.20 |  | 21.13 | 20.66 | 21.59 |
|  |  | 50 | 13.11 | 12.76 | 13.44 |  | 14.28 | 13.93 | 14.61 |
|  |  | 60 | 7.00 | 6.82 | 7.17 |  | 7.57 | 7.39 | 7.73 |
|  | Hispanic  (men: n=697, women: n=641) | 30 | 29.20 | 28.49 | 29.94 |  | 28.39 | 27.67 | 29.10 |
|  |  | 40 | 21.53 | 20.98 | 22.10 |  | 21.34 | 20.75 | 21.90 |
|  |  | 50 | 14.26 | 13.86 | 14.66 |  | 14.44 | 14.01 | 14.83 |
|  |  | 60 | 7.44 | 7.25 | 7.63 |  | 7.66 | 7.47 | 7.84 |
|  | Non-Black, Non-Hispanic  (men: n=1,895, women: n=1,669) | 30 | 32.60 | 32.16 | 32.97 |  | 29.83 | 29.40 | 30.20 |
|  |  | 40 | 24.21 | 23.86 | 24.50 |  | 22.39 | 22.02 | 22.70 |
|  |  | 50 | 16.05 | 15.78 | 16.28 |  | 15.08 | 14.79 | 15.33 |
|  |  | 60 | 8.19 | 8.06 | 8.31 |  | 7.90 | 7.74 | 8.02 |
| Episodic, Before 40 | Black  (men: n=150, women: n=211) | 30 | 17.48 | 16.04 | 18.92 |  | 23.50 | 22.09 | 24.74 |
|  |  | 40 | 12.91 | 11.83 | 14.20 |  | 17.65 | 16.49 | 18.66 |
|  |  | 50 | 9.01 | 8.12 | 9.91 |  | 12.13 | 11.30 | 12.84 |
|  |  | 60 | 5.73 | 5.20 | 6.23 |  | 6.93 | 6.53 | 7.24 |
|  | Hispanic  (men: n=73, women: n = 125) | 30 | 24.34 | 20.25 | 27.01 |  | 23.66 | 21.26 | 25.44 |
|  |  | 40 | 18.06 | 15.40 | 19.94 |  | 17.88 | 16.24 | 19.17 |
|  |  | 50 | 12.13 | 10.68 | 13.43 |  | 12.35 | 11.29 | 13.20 |
|  |  | 60 | 6.82 | 6.23 | 7.36 |  | 7.07 | 6.63 | 7.42 |
|  | Non-Black, Non-Hispanic  (men: n=157, women=279) | 30 | 26.52 | 24.81 | 27.90 |  | 24.44 | 23.12 | 25.43 |
|  |  | 40 | 19.70 | 18.27 | 20.83 |  | 18.44 | 17.36 | 19.28 |
|  |  | 50 | 13.14 | 12.03 | 14.01 |  | 12.62 | 11.82 | 13.26 |
|  |  | 60 | 7.18 | 6.66 | 7.59 |  | 7.04 | 6.61 | 7.36 |
| Episodic, Age 40 | Black  (men: n=83, women: n=121) | 30 | 15.51 | 13.76 | 17.37 |  | 18.73 | 16.98 | 20.46 |
|  |  | 40 | 10.69 | 9.23 | 12.27 |  | 13.63 | 12.10 | 15.08 |
|  |  | 50 | 7.38 | 6.07 | 8.62 |  | 9.25 | 7.79 | 10.39 |
|  |  | 60 | 5.01 | 3.72 | 5.98 |  | 5.48 | 4.10 | 6.32 |
|  | Hispanic  (men: n=44, women: n=53) | 30 | 17.42 | 6.44 | 21.75 |  | 18.15 | 6.08 | 22.56 |
|  |  | 40 | 12.55 | 4.23 | 15.78 |  | 13.59 | 6.55 | 16.82 |
|  |  | 50 | 8.84 | 2.13 | 11.03 |  | 9.42 | 3.25 | 11.53 |
|  |  | 60 | 5.79 | 1.05 | 7.04 |  | 5.63 | 0.96 | 6.87 |
|  | Non-Black, Non-Hispanic  (men: n=85, women: n=140) | 30 | 22.95 | 20.67 | 24.88 |  | 19.81 | 17.55 | 21.57 |
|  |  | 40 | 16.29 | 14.26 | 17.93 |  | 14.29 | 12.59 | 15.74 |
|  |  | 50 | 10.72 | 8.93 | 12.02 |  | 9.49 | 8.02 | 10.73 |
|  |  | 60 | 6.33 | 5.02 | 7.12 |  | 5.36 | 3.70 | 6.38 |
| Episodic, Age 50 | Black  (men: n=65, women: n=120) | 30 | 15.55 | 10.21 | 17.63 |  | 18.24 | 15.98 | 19.94 |
|  |  | 40 | 9.21 | 4.75 | 11.01 |  | 12.48 | 10.77 | 13.93 |
|  |  | 50 | 5.33 | 2.10 | 6.69 |  | 7.98 | 6.64 | 9.05 |
|  |  | 60 | 3.25 | 0.67 | 4.35 |  | 4.59 | 3.31 | 5.46 |
|  | Hispanic  (men: n=49, women: n=56) | 30 | 17.74 | 15.39 | 19.71 |  | 16.03 | 9.69 | 18.86 |
|  |  | 40 | 10.81 | 9.15 | 12.50 |  | 10.70 | 6.25 | 12.94 |
|  |  | 50 | 6.40 | 5.01 | 7.70 |  | 6.62 | 2.98 | 8.21 |
|  |  | 60 | 3.93 | 2.29 | 4.95 |  | 3.76 | 0.90 | 4.80 |
|  | Non-Black, Non-Hispanic  (men: n=103, women: n=177) | 30 | 19.91 | 18.41 | 21.41 |  | 22.27 | 20.57 | 23.52 |
|  |  | 40 | 12.24 | 10.85 | 13.64 |  | 15.32 | 14.05 | 16.42 |
|  |  | 50 | 6.89 | 5.69 | 8.15 |  | 9.44 | 8.25 | 10.38 |
|  |  | 60 | 3.83 | 2.58 | 4.82 |  | 4.94 | 3.84 | 5.65 |
| Persistent High Symptom | Black  (men: n=22, women: n=68) | 30 | 10.26 | 5.02 | 12.47 |  | 11.50 | 5.37 | 13.78 |
|  |  | 40 | 7.55 | 4.62 | 9.70 |  | 8.08 | 3.20 | 9.89 |
|  |  | 50 | 5.97 | 3.71 | 7.74 |  | 5.80 | 1.83 | 7.29 |
|  |  | 55/60* | 5.48 | 3.24 | 7.06 |  | 4.36 | 0.59 | 5.44 |
|  | Hispanic  (men: n=20, women: n=52) | 30 | 12.93 | 3.25 | 15.79 |  | 10.33 | 4.24 | 12.84 |
|  |  | 40 | 9.67 | 4.46 | 12.02 |  | 7.26 | 4.67 | 9.10 |
|  |  | 50 | 7.78 | 4.76 | 9.55 |  | 5.34 | 3.97 | 6.70 |
|  |  | 55/60* | 7.15 | 4.20 | 8.61 |  | 4.06 | 2.33 | 5.19 |
|  | Non-Black, Non-Hispanic  (men: n=31, women: n=84) | 30 | 15.74 | 7.97 | 18.06 |  | 14.69 | 12.53 | 16.49 |
|  |  | 40 | 11.26 | 7.21 | 13.47 |  | 10.32 | 8.57 | 11.88 |
|  |  | 50 | 8.32 | 5.33 | 10.18 |  | 6.82 | 5.28 | 8.08 |
|  |  | 55/60* | 7.26 | 4.65 | 8.76 |  | 4.40 | 2.74 | 5.50 |

WLE, Working Life Expectancy. * Highest estimate is 55 years for men, 60 years for women

**Supplemental Figure 1.2. Estimated Working Life Expectancy by Gender, Race/Ethnicity, and Depressive Symptom Trajectory
Panel A. Men**

**
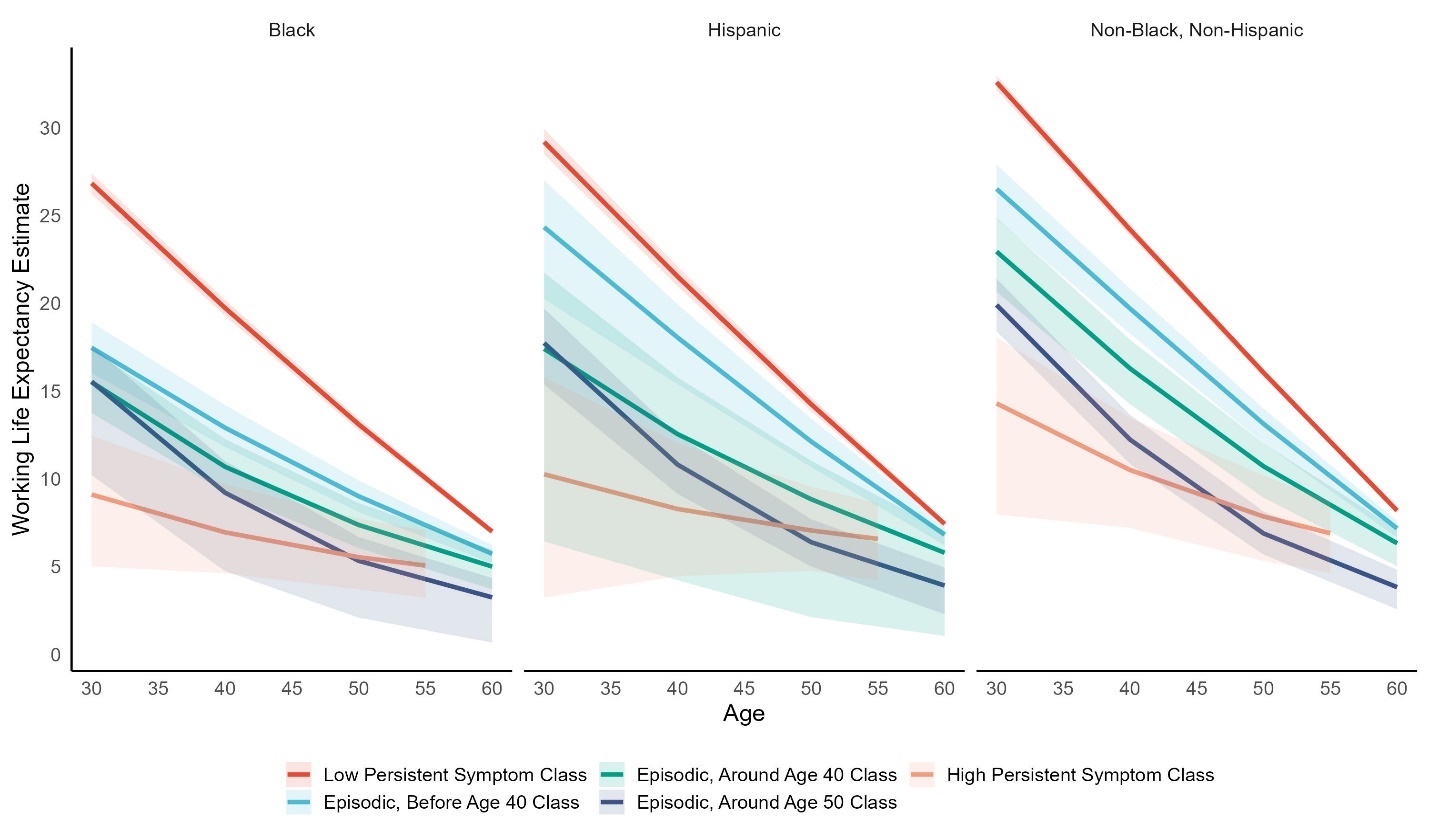
**

**Panel B. Women**

**
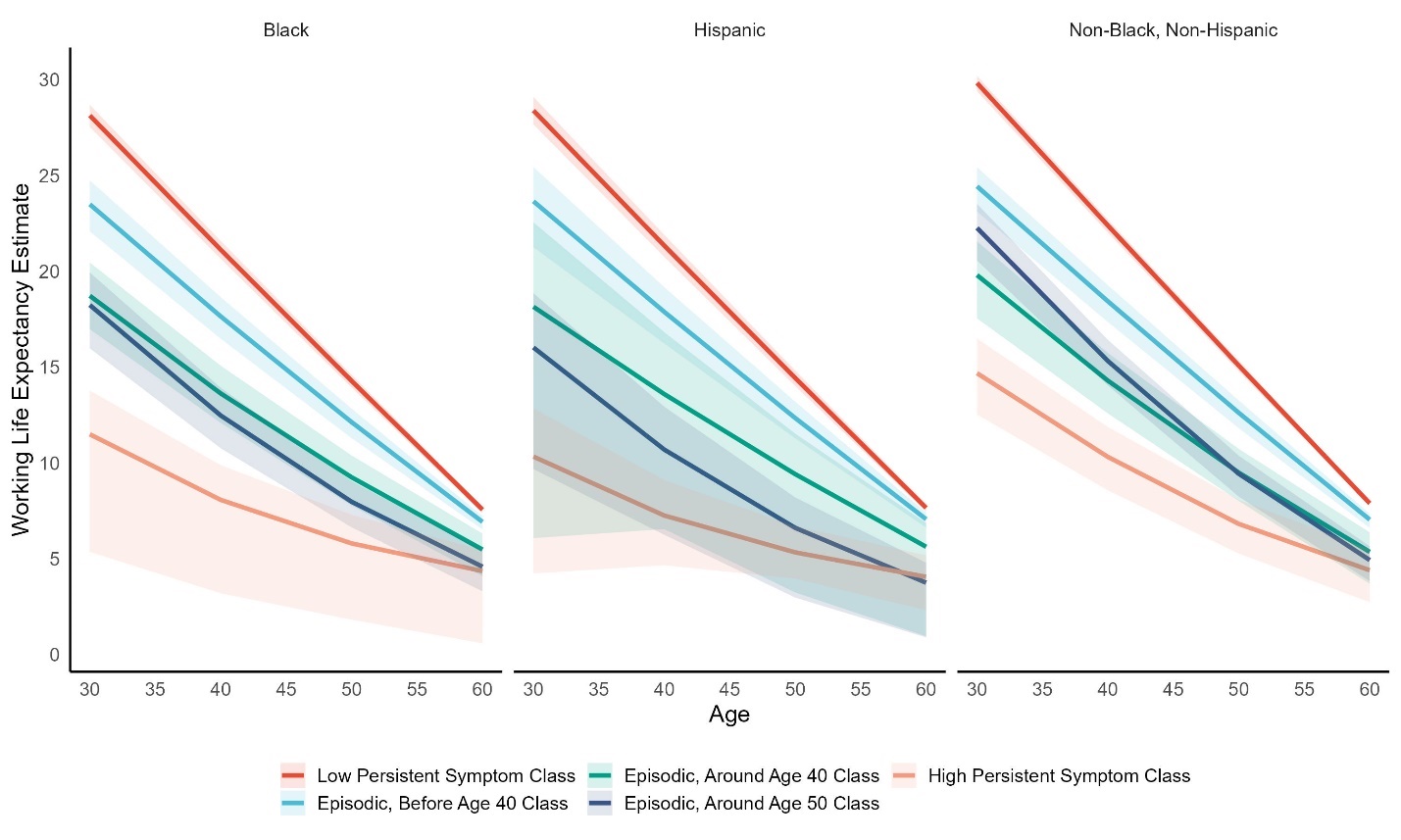
**

**Supplemental Table 1.6. Working Life Expectancy Between Ages 30 to 60, Stratified by Gender, Highest Level of Education at Age 30, and Depressive Symptom Trajectory**

|  |  |  | Men | | |  | Women | | |
| --- | --- | --- | --- | --- | --- | --- | --- | --- | --- |
| Trajectory Class | Education Level | Age | WLE | 95%CI | |  | WLE | 95%CI | |
| Persistent Low Symptom | Less than High School (men: n = 499, women: n = 318) | 30 | 25.01 | 24.14 | 25.82 |  | 20.93 | 19.83 | 21.90 |
|  |  | 40 | 18.34 | 17.64 | 18.95 |  | 15.77 | 14.85 | 16.53 |
|  |  | 50 | 12.16 | 11.67 | 12.59 |  | 10.89 | 10.22 | 11.41 |
|  |  | 60 | 6.52 | 6.27 | 6.72 |  | 6.20 | 5.87 | 6.44 |
|  | High School Diploma  (men: n=2,202, women: n=2,044) | 30 | 30.06 | 29.63 | 30.45 |  | 29.50 | 29.08 | 29.88 |
|  |  | 40 | 22.18 | 21.79 | 22.54 |  | 22.14 | 21.76 | 22.48 |
|  |  | 50 | 14.67 | 14.38 | 14.96 |  | 14.91 | 14.62 | 15.18 |
|  |  | 60 | 7.59 | 7.43 | 7.74 |  | 7.82 | 7.67 | 7.97 |
|  | College Degree  (men: n=728, women: n=684) | 30 | 35.15 | 34.61 | 35.69 |  | 31.90 | 31.28 | 32.42 |
|  |  | 40 | 26.18 | 25.79 | 26.59 |  | 23.94 | 23.46 | 24.36 |
|  |  | 50 | 17.38 | 17.11 | 17.64 |  | 16.06 | 15.72 | 16.35 |
|  |  | 60 | 8.78 | 8.65 | 8.90 |  | 8.27 | 8.11 | 8.41 |
| Episodic, Before 40 | Less than High School  (men: n = 86, women: n = 112) | 30 | 16.79 | 14.83 | 18.53 |  | 17.01 | 14.98 | 18.53 |
|  |  | 40 | 12.45 | 10.94 | 13.82 |  | 13.04 | 11.40 | 14.28 |
|  |  | 50 | 8.71 | 7.63 | 9.67 |  | 9.37 | 8.19 | 10.23 |
|  |  | 60 | 5.58 | 4.99 | 6.12 |  | 5.94 | 5.43 | 6.34 |
|  | High School Diploma  (men: n=237, women: n=420) | 30 | 23.73 | 22.33 | 24.98 |  | 25.35 | 24.22 | 26.29 |
|  |  | 40 | 17.38 | 16.25 | 18.59 |  | 19.06 | 18.11 | 19.88 |
|  |  | 50 | 11.63 | 10.71 | 12.54 |  | 13.01 | 12.21 | 13.68 |
|  |  | 60 | 6.62 | 6.14 | 7.04 |  | 7.22 | 6.83 | 7.54 |
|  | College Degree  (men: n=24, women: n=65) | 30 | 29.95 | 22.05 | 34.15 |  | 28.12 | 21.17 | 30.72 |
|  |  | 40 | 22.82 | 17.90 | 25.59 |  | 21.47 | 18.90 | 23.12 |
|  |  | 50 | 15.44 | 12.81 | 17.03 |  | 14.56 | 13.12 | 15.56 |
|  |  | 60 | 8.11 | 7.39 | 8.64 |  | 7.79 | 7.28 | 8.17 |
| Episodic, Age 40 | Less than High School  (men: n=68, women: n=61) | 30 | 12.63 | 0.78 | 15.56 |  | 12.49 | 4.75 | 15.31 |
|  |  | 40 | 8.79 | 0.40 | 11.14 |  | 9.26 | 5.01 | 11.23 |
|  |  | 50 | 6.20 | 0.15 | 8.07 |  | 6.58 | 4.75 | 7.92 |
|  |  | 60 | 4.46 | 0.02 | 5.76 |  | 4.25 | 2.76 | 5.28 |
|  | High School Diploma  (men: n=122, women: n=208) | 30 | 19.01 | 4.77 | 22.89 |  | 20.81 | 19.06 | 22.18 |
|  |  | 40 | 13.75 | 6.53 | 16.26 |  | 15.17 | 13.76 | 16.42 |
|  |  | 50 | 9.37 | 6.67 | 10.99 |  | 10.22 | 8.91 | 11.25 |
|  |  | 60 | 5.74 | 4.03 | 6.63 |  | 5.93 | 4.68 | 6.60 |
|  | College Degree  (men: n=12, women: n=30) | 30 | 32.26* | 8.33 | 35.63 |  | 24.62 | 9.11 | 30.48 |
|  |  | 40 | 23.33* | 9.52 | 26.26 |  | 18.53 | 10.23 | 22.65 |
|  |  | 50 | 14.62 | 7.48 | 17.29 |  | 12.57 | 7.91 | 15.13 |
|  |  | 60 | 7.11 | 3.89 | 8.87 |  | 6.88 | 3.06 | 8.04 |
| Episodic, Age 50 | Less than High School  (men: n=62, women: n=55) | 30 | 13.01 | 0.07 | 16.59 |  | 12.44 | 0.13 | 15.90 |
|  |  | 40 | 7.61 | 0.01 | 10.16 |  | 8.26 | 0.01 | 10.94 |
|  |  | 50 | 4.53 | 0.00 | 6.22 |  | 5.26 | 0.00 | 7.29 |
|  |  | 60 | 2.94 | 0.00 | 4.27 |  | 3.24 | 0.00 | 4.65 |
|  | High School Diploma  (men: n=127, women: n=245) | 30 | 19.48 | 18.07 | 20.86 |  | 20.63 | 18.60 | 21.88 |
|  |  | 40 | 11.88 | 10.70 | 13.20 |  | 14.10 | 12.55 | 15.24 |
|  |  | 50 | 6.62 | 5.52 | 7.70 |  | 8.75 | 7.70 | 9.69 |
|  |  | 60 | 3.69 | 2.55 | 4.55 |  | 4.73 | 3.62 | 5.43 |
|  | College Degree  (men: n=13, women: n=36) | 30 | 24.71 | 6.82 | 30.85 |  | 23.83 | 15.17 | 27.32 |
|  |  | 40 | 16.47 | 3.37 | 21.66 |  | 16.59 | 9.38 | 19.52 |
|  |  | 50 | 9.56 | 1.38 | 13.19 |  | 10.34 | 5.23 | 12.47 |
|  |  | 60 | 4.79 | 0.20 | 6.57 |  | 5.51 | 1.82 | 6.70 |
| Persistent High Symptom | Less than High School  (men: n=31, women: n=66) | 30 | 12.25 | 4.05 | 16.02 |  | 8.67 | 7.01 | 10.09 |
|  |  | 40 | 9.05 | 4.90 | 12.05 |  | 6.07 | 4.99 | 7.10 |
|  |  | 50 | 6.76 | 4.95 | 8.78 |  | 4.35 | 3.42 | 5.30 |
|  |  | 60 | 6.76 | 4.95 | 8.78 |  | 3.38 | 2.03 | 4.31 |
|  | High School Diploma ^#^  (men: n=36**, women: n=124) | 30 | 12.90 | 9.38 | 15.96 |  | 15.03 | 9.76 | 16.92 |
|  |  | 40 | 9.78 | 7.21 | 12.26 |  | 10.49 | 7.02 | 12.11 |
|  |  | 50 | 7.72 | 5.32 | 9.88 |  | 7.13 | 5.41 | 8.42 |
|  |  | 55/60 | 6.90 | 4.84 | 8.68 |  | 4.80 | 3.52 | 5.69 |
|  | College Degree  (men: n=3, women: n=9) | 30 | NA | NA | NA |  | 10.47 | 2.76 | 15.94 |
|  |  | 40 | NA | NA | NA |  | 8.02 | 1.43 | 11.88 |
|  |  | 50 | NA | NA | NA |  | 5.54 | 0.44 | 8.32 |
|  |  | 60 | NA | NA | NA |  | 3.78 | 0.14 | 5.65 |

NA, not applicable due to small sample size. * point estimate value, ^#^ highest WLE value is 55 years for men, and 60 years for women, ** for men in the persistent high symptom group, the sample size also includes the 3 participants who had a college degree.

**Supplemental Figure 1.3. Estimated Working Life Expectancy by Gender, Education Level at Age 30, and Depressive Symptom Trajectory
Panel A. Men

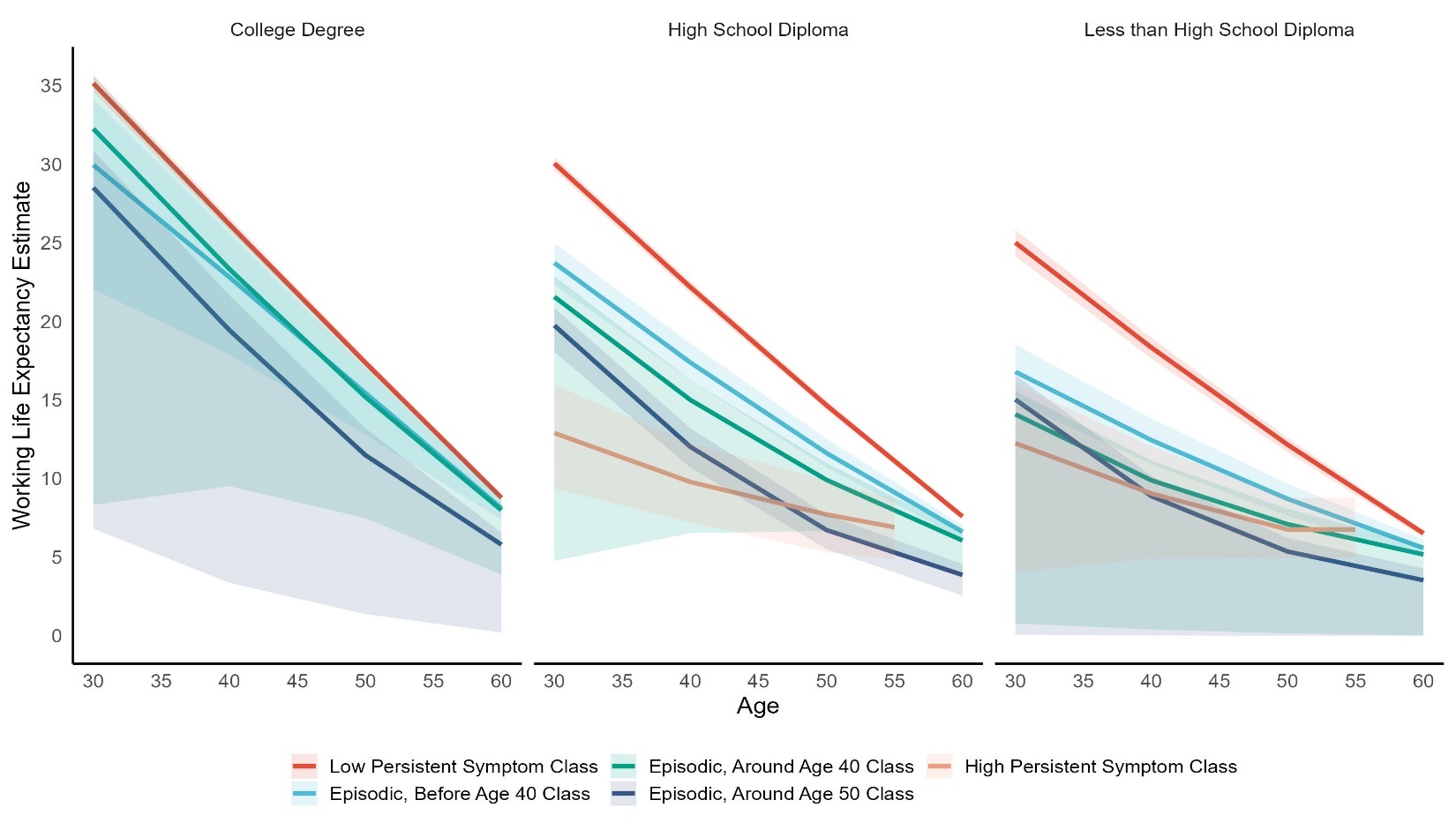
**

**Panel B Women


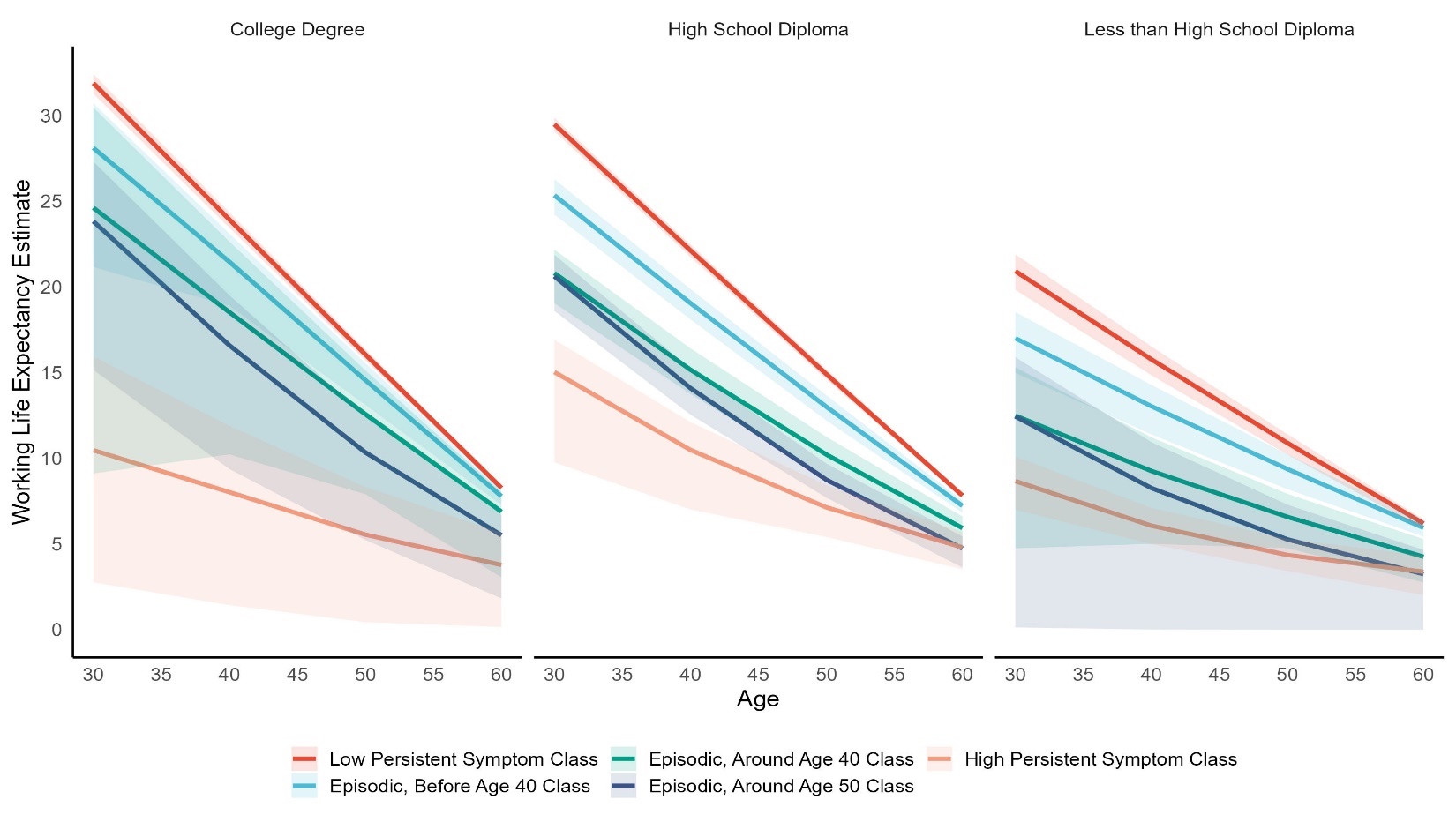
**
