# Supplementary material for: The working life expectancy of American adults experiencing depression
Source: Soc Psychiatry Psychiatr Epidemiol. 2023 Sep 7;59(6):1013–27. doi: 10.1007/s00127-023-02547-4 (PMC11116182; doi:10.1007/s00127-023-02547-4)

**The Working Life Expectancy of American Adults Experiencing Depression**

**Supplemental File 2**

**Sociodemographic, Health, and Labor-Related Profile of NLSY79 Participants, by Depressive Symptom Latent Class**

**Description of Variables**

Guided by a life course conceptual framework, we explored factors that may influence depression and labor market experiences. We explored baseline factors measured in 1979, which include participant birth year, gender, country of birth, state region, residing in a rural location, parental living and working status at age fourteen, family size, total net family income, family poverty status, and highest grade completed. As risky health behaviors may influence the risk of depression and poor labor market participation, we also explored markers of illegal activity and alcohol use.

To understand the sociodemographic profile of the cohort over time, sociodemographic factors associated with depression and labor market trajectories at age 20, 30, 40, and 50 were assessed.[1–6] Time-varying demographic characteristics were state region, urban residence, highest level of education, marital status, family size, number of children, and age of youngest child.

The following health-related factors [7–11] from the Health at 40 module were included: 1) if a participants mother and father were still living; 2) if a participant reported ever seeing a physician for depression; 3) if a participant self-reported ever experiencing depression; 4) if a participant had physical health conditions (including high blood pressure, diabetes, cancer, lung problems, heart problems/failure, stroke, or arthritis) diagnosed by a physician; and 5) the SF12 Physical Health and Mental Health Scores.[12] These questions were also included in the health at 50 module, as well as physician diagnosis of a mental health condition apart from depression.

Labor force-related factors that may provide context to the depressive symptom trajectories and working life expectancy metrics were examined. These factors included the number of jobs a participant held in the past year; the total number of hours (annually) a participant worked in the past year, the amount of annual employment income, and annual measures of receipt and amount of welfare, social security, and disability income. Earning values were inflated to 2022 USD.[13] For welfare, social security, or disability income variables, amounts reported reflect a combined amount for both the respondent and/or their spouse. We report labor factors both by age, and in some cases by year (1992, reflecting the first year of the CES-D-SF measurement, and 2016).

**Analysis**

Once the most appropriate depressive symptom latent trajectory model was selected, differences in baseline demographic and health factors by trajectory class were explored using Chi-square tests for categorical variables and ANOVA tests for continuous variables. The distribution of time-varying demographic and health factors are presented for ages 20, 30, 40, and 50. For respondents who turned 30, 40, or 50 years old during a year where survey assessment was not completed, we report on their demographic and health information from the next survey cycle. For example, if a participant turned 40 in 2003 (a year where no survey occurred), we obtained their educational information from the 2004 survey cycle year.

Descriptive statistics for the labor-related variables were calculated annually, starting at age 18-20, until the end of follow-up. We highlight frequency of income source or labor market indicator, and average or median values of the variables listed on page 1.

**Profile of Depressive Symptom Trajectory Classes**

**Baseline (1979-1980) Characteristics (Supplemental Table 2.1)**

In the persistent low symptom trajectory (n=6,838), the average age of participants was 17.5 years (SD=2.2 years). About 49% of participants were men, and half of participants in this class were Non-Black, Non-Hispanic. In 1979, roughly 7% of participants in the low symptom class reported being born outside of the United States. Most participants lived in an urban area or Southern state region. Most participants were still completing elementary or secondary school, with ~10% having completed one year of college.

Most participants reported living in a family with 3 or more people. Less than 2% reported that their biological mother was no longer living, and 8% reported that their biological father was no longer living. Around 8% of participants reported being married. Of the participants who were younger than 18 years old in 1980, ~27% reported never using alcohol in the past year (14% of the entire class), and 22% reported using alcohol 2-10 times in the past year (11% of the entire class). In 1980, about half of participants reported never using marijuana, with 13% reporting using it more than 50 times. ~77% reported never using hard drugs within the past 12 months.

In the episodic, before age 40 trajectory (n=995), the average age of participants was 17.5 years (SD=2.2 years). 62% of participants in this class were women, 36% reported Black race/ethnicity, ~20% reported Hispanic race/ethnicity, and ~44% were Non-Black, Non-Hispanic (NBNH). Most participants reported living in a southern state and in an urban (82%) region. 9% of participants reported that their birth father was no longer living. Distributions for family size, number of children, marital status, highest level of education completed, if their birth mother was still living, and substance/police interactions were similar to the persistent low symptom trajectory class.

In the episodic around age 40 class (n=526), the average age of participants was 17.6 years (SD=2.1 years). Roughly 60% of participants in this class were women. 39% of participants in this class were Black, 18% were Hispanic, and 43% were NBNH. The remaining distribution of baseline factors were similar to the episodic, before age 40 trajectory.

In the episodic, around age 50 trajectory class (n=570), the average age of participants was 17.6 years (SD=2.4 years), ~62% were women, 33% were Black, 18% were Hispanic, and 49% were NBNH. Roughly 11% of participants were married, with ~15% reporting having at least one child. The distribution of other variables was similar to the previous trajectory groups.

In the final trajectory, defined by persistent depressive symptoms (n=277), the average age of participants was 17.8 years (SD=2.2) and nearly 75% were women. 33% of participants were Black, 26% were Hispanic, and 42% were NBNH. About 15% of responders were married, with 18% reported having at least 1 child. This trajectory group had the highest proportion of participants ever stopped by the police (20.2% vs. 16.6-19.5% in the other trajectory groups). The distribution of other variables was similar to the previous trajectory groups.

**Sociodemographic Characteristics: Ages 20 – 50**

Supplemental Table 2.2 highlights the distribution of the following variables at age 20, 30, 40, and 50: state region, rural region, highest level of education, family size, marital status, and number of children. Supplemental Table 2.3 highlights the correlation for each factor by age, stratified by depressive symptom trajectory.

*Geography.* Among all trajectory groups, the distribution of state region was similar across each decade were highly correlated (r=0.78 to 0.95). The distribution of rural/urban region was also similar: at age 20 and 30, ~75% of participants resided in an urban region, decreasing to ~60-65% at age 40 and 50.

*Highest Level of Education.* At age 30, ~20% of the persistent low symptom trajectory class reported having a college degree as their highest level of education, which remained stable in their 40s and 50s. Among the episodic before age 40 trajectory group, only 9% of respondents reported having a college degree, increasing to 11% at age 40. This was similar among the other two episodic trajectories. Among the persistent high symptom trajectory, around 5% of participants reported having a college degree at age 30, 40 and 50.

*Marital Status.* At age 20, a greater proportion of participants in the persistent high depressive symptom trajectory were married (23%) compared to the other trajectories (12-19%). At age 30, ~53% of participants in the persistent low symptom trajectory were married, ~40-43% of participants in the episodic symptom trajectories were married, and 34% of participants in the persistent high symptom trajectory were married. At ages 40 and 50, 45-50% of the persistent low symptom class remained married and ~35% of the episodic, before age 40 trajectory remained married. Among the episodic at age 40 trajectory, 35% of participants remained married at age 40, decreasing to 29% at age 50. Among the episodic at age 50 trajectory, ~43% remained married at age 40, decreasing to 39% at age 50. Among the persistent high symptom trajectory, ~26% were married at age 40 and 22% were married at age 50.

*Family Size and Number of Children.* At age 20 and 30, most participants across all 5 trajectory groups lived in a household with at least 3 individuals. At age 40 and 50, this distribution shifted, with a greater proportion of participants living in a household with 1 – 3 individuals. At age 50, ~18% of the persistent low symptom trajectory, 21% of the episodic before age 40 trajectory, 29% of the episodic at age 40, 27% of the episodic at age 50, and 26% of the persistent high symptom trajectory lived alone.

At age 20, a greater proportion of participants in the low symptom trajectory reported not living with any biological or adopted children (86%) compared to those in the episodic trajectories (~70-75%) or persistent high symptom trajectory (66%). By age 30, over half of the persistent low symptom trajectory, ~60% of participants in the episodic trajectories, and 70% of the persistent high symptom group reported having one child living with them. By age 50, most participants had at least one child still living with them.

**Health Characteristics: Age 40 and 50 (Supplemental Table 2.4)**

Among the persistent low symptom trajectory, when participants were 40 years old, roughly 38% reported that their father had passed away and 19% reported that their mother had passed away. Roughly 27% of participants reported having at least one other health condition. This trajectory group reported the highest scores on the SF12 Physical and Mental Health scales. This was similar to what participants reported in the health at 50 module.

Among the episodic, before age 40 trajectory, 42% reported that their father had passed away and 21% reported that their mother had passed away. Nearly 36% reported having 2 or more chronic health conditions. The average scores on the SF12 physical component were ~2 points lower than the persistent low symptom trajectory group and the average scores on the SF mental component were ~3 points lower. At age 40, 20% reported that their emotional problems caused them to accomplish less in the past month. When measured at age 50, the scores on the SF12 Physical and Mental Health Component scores were similar to when aged 40.

Among the episodic at age 40 trajectory, 42% reported that their father had passed away and 25% reported that their mother had passed away. Roughly half of respondents reported having 2 or more chronic health conditions. The average scores on the SF12 physical component were ~5 points lower than the persistent low symptom trajectory group and the average scores on the SF mental component were ~14 points lower. At age 40, 48% reported that their emotional problems caused them to accomplish less in the past month. When age 50, the scores on the SF12 Physical Component score was slightly lower and scores on the SF12 Mental Health component were slightly higher (50.41).

Among the episodic at age 50 trajectory, 40% reported that their father had passed away and 21% reported that their mother had passed away. Forty-four percent of respondents reported having 2 or more chronic health conditions. The average scores on the SF12 physical and mental health component were ~3-4 points lower than the persistent low symptom trajectory group. 27% reported that their emotional problems caused them to accomplish less in the past month. When age 50, the scores on the SF12 Physical Component score were 12 points lower and ~20 points lower on the SF12 Mental Component.

Lastly, among the persistent high symptom trajectory, 46% reported that their father had passed away and 30% reported that their mother had passed away. About 60% reported having 2 or more chronic health conditions. The average scores on the SF12 physical component were ~8 points lower than the low symptom trajectory group and the average scores on the SF mental component were ~20 points lower. At age 40, ~62% reported that their emotional problems caused them to accomplish less in the past month. When measured at age 50, the scores on the SF12 Mental Health Component score were similar to when aged 40, but the median SF12 physical score had decreased an additional 12 points (~48 at age 40 and ~37 at age 50).

**Labor and Income Indicators, Overview (Figures 2.1 – 2.3)**

*Overview.* Between ages 20 to 50, ~2-8% of participants in the persistent low symptom trajectory reported receiving some sort of welfare income. Among the episodic symptoms before age 40 class, ~17% of participants reported receiving welfare income at age 20, 21% at age 30, 6% at age 40, and 8% at age 50. Among the episodic symptom at age 40 and 50 symptom trajectories, similar trends were seen. Among the persistent high symptom trajectory, there were 30% of participants at age 20 who reported receiving welfare income, which increased to 43% at age 30, 16% at age 40, and 18% at age 50.

Supplemental Figure 1 shows the median annual work hours between ages 18 to 59 among each depressive symptom trajectory. Description of trends by depressive symptom trajectory is described in the text below.

Supplemental Figure 2 shows the average median employment income values among depressive symptom trajectory between ages 18 to 61. Among the persistent low symptom trajectory class, average earnings reach ~$51,000 USD at age 60. Among the episodic, before age 40 class, average earnings reach ~$19,000 USD at age 60. For the two remaining trajectories, average earnings reach ~$12,000-14,000 at age 60; however, the two episodic trajectories have a steeper earning slope between ages 20 to 55 compared to the persistent high symptom trajectory. The persistent high symptom trajectory had average earnings of ~$20,000 at age 60. While trajectory shapes are similar, median earnings over the working years are deflated for all trajectories, particularly the persistent high symptom trajectory.

Supplemental Figure 3 shows the median welfare income values between ages 18 to 61. Generally, the amount of welfare income received was similar between trajectories until the mid-40s. After this time, the persistent high symptom trajectory group was receiving ~$2,000-$3,000 more than the low symptom trajectory, and the episodic before age 40 and episodic at age 40 showed an upward trajectory of welfare income.

**Labor and Income Indicators, by Depressive Symptom Trajectory**

*Persistent Low Symptom Trajectory.* Between 1979 to 2016, most participants did not receive disability income (66%) or did only once (17%) or twice (7%). In 1992, the year with the first CES-D-SF measurement, most participants reported having two jobs (64%) and working 2000 – 2499 hours per year (Supplemental Figure 2.1). Roughly 20% of participants worked in managerial or professional specialty occupations. This was similar in 1994 until 2016. Between age 20 to 50, ~1-2% of participants in the low symptom trajectory were receiving social security income.

*Episodic, Before Age 40 Trajectory.* Between 1979 to 2016, most participants had received disability income at least once, with the majority receiving it one to four times. In 1992, ~20% reported having no job in the past year, 13% reported one job, and 51% reported having two jobs. Roughly 26% worked 2000 to 2499 hours per year and 14% were employed in an administrative support/clerical or service occupation. In 2016, 24% of respondents reported having no job in the past year, 42% reported having one job, and 7% reported having two jobs. For those working, most worked ~2000 to 2499 hours per year. Among the episodic before age 40 trajectory, ~5% reported receiving social security income at age 20, increasing to ~6% at age 30, and decreased to 2-3% age 40 and 50.

*Episodic, Age 40 Trajectory.* Between 1979 to 2016, most participants had received disability income at least once, with the majority receiving it one to four times. In 1992, ~26% reported having no job in the past year,10% reported one job, and 48% reported having two jobs. Roughly 26% worked 2000 to 2499 hours per year and 11% were employed in an administrative support/clerical or service occupation. In 2016, 31% of respondents reported having no job in the past year, 35% reported having one job, and 8% reported having two jobs. For those working, most worked ~2000 to 2499 hours per year. Among the episodic at age 40 trajectory, ~5% of participants reported receiving social security income at age 20, which increased to 10% at age 30, and decreased to ~6% at age 40 and 50.

Episodic, *Age 50 Trajectory.* Between 1979 to 2016, most participants had received disability income at least once, with the majority receiving it one to four times. In 1992, ~21% reported having no job in the past year, 10% reported one job, and 54% reported having two jobs. Roughly 31% worked 2000 to 2499 hours per year, and 16% were employed in an administrative support/clerical or service occupation. In 2016, most respondents reported having no job in the past year (46%), followed by one job (32%) and two jobs (7%). For those working, most worked ~2000 to 2499 hours per year. Among the episodic around age 50 trajectory, at age 20 ~3% of participants reported receiving social security income, which increased to 5% at age 30, 4% at age 40, and 7% at age 50.

*Persistent high symptom Trajectory.* Between 1979 to 2016, most participants in the persistent high symptom trajectory group had received disability income at least once, with the majority receiving it one to five times. In 1992, ~37% reported having no job in the past year, 16% reported one job, and 31% reported having two jobs. Roughly 15% worked 2000 to 2499 hours per year and were employed in a service occupation. Among the persistent high symptom trajectory, roughly 6% reported receiving social security income at age 20, increasing to 14% at age 30, 11% at age 40, and 12% at age 50. Similar trends were seen for disability income.

**References**

1. Sareen J, Afifi TO, McMillan KA, Asmundson GJG (2011) Relationship between household income and mental disorders: Findings from a population-based longitudinal study. Arch Gen Psychiatry 68:419–427. https://doi.org/10.1001/archgenpsychiatry.2011.15

2. Ettner SL, Frank RG, Kessler RC (1997) The Impact of Psychiatric Disorders on Labor Market Outcomes. ILR Review 51:64–81

3. Kessler RC, Merikangas KR, Wang PS (2008) The prevalence and correlates of workplace depression in the national comorbidity survey replication. J Occup Environ Med 50:381–390. https://doi.org/10.1097/JOM.0b013e31816ba9b8

4. Breslau J, Lane M, Sampson N, Kessler RC (2008) Mental disorders and subsequent educational attainment in a US national sample. J Psychiatr Res 42:708–716. https://doi.org/10.1016/j.jpsychires.2008.01.016

5. Kessler RC, Berglund P, Demler O, et al (2005) Lifetime Prevalence and Age-of-Onset Distributions of DSM-IV Disorders in the National Comorbidity Survey Replication. Arch Gen Psychiatry 62:593–602. https://doi.org/10.1001/archpsyc.62.6.593

6. Patel V, Burns JK, Dhingra M, et al (2018) Income inequality and depression: a systematic review and meta-analysis of the association and a scoping review of mechanisms. World Psychiatry 17:76–89

7. Patel V, Burns JK, Dhingra M, et al (2018) Income inequality and depression: a systematic review and meta-analysis of the association and a scoping review of mechanisms. World Psychiatry 17:76–89

8. Farella Guzzo M, Gobbi G (2021) Parental Death During Adolescence: A Review of the Literature. Omega (United States). https://doi.org/10.1177/00302228211033661/ASSET/IMAGES/LARGE/10.1177_00302228211033661-FIG2.JPEG

9. Bélanger SM, Stene-Larsen K, Magnus P, et al (2022) Employment status and bereavement after parental suicide: a population representative cohort study. BMJ Open 12:e064379. https://doi.org/10.1136/BMJOPEN-2022-064379

10. Barnes GE, Prosen H (1985) Parental Death and Depression. J Abnorm Psychol 94:64–69. https://doi.org/10.1037/0021-843X.94.1.64

11. Kessler RC (2012) The costs of depression. Psychiatric Clinics 35:1–14. https://doi.org/10.1016/j.psc.2011.11.005

12. Hays RD, Sherbourne CD, Mazel RM (1993) The RAND 36‐item health survey 1.0. Health Econ 2:217–227. https://doi.org/10.1002/hec.4730020305

13. US Inflation Calculator (2023) Consumer Price Index Data from 1913 to 2023. https://www.usinflationcalculator.com/inflation/consumer-price-index-and-annual-percent-changes-from-1913-to-2008/. Accessed 31 Jul 2020

**Supplemental Tables**

**Supplemental Table 2.1. Baseline Demographics, by Depressive Symptom Trajectory Group**

|  | Overall | Persistent Low Depressive Symptoms | Episodic, Before Age 40 | Episodic, Around Age 40 | Episodic, Around Age 50 | Persistent High Depressive Symptoms | p |
| --- | --- | --- | --- | --- | --- | --- | --- |
| N | 9206 | 6838 | 995 | 526 | 570 | 277 |  |
| **Age in 1979 (mean (SD))** | 17.50 (2.23) | 17.48 (2.24) | 17.49 (2.19) | 17.62 (2.13) | 17.60 (2.38) | 17.76 (2.23) | 0.133 |
| **Birth year (%)** |  |  |  |  |  |  | 0.011 |
| 1957 | 947 (10.3) | 703 (10.3) | 87 (8.7) | 46 (8.7) | 80 (14.0) | 31 (11.2) |  |
| 1958 | 988 (10.7) | 716 (10.5) | 111 (11.2) | 56 (10.6) | 70 (12.3) | 35 (12.6) |  |
| 1959 | 1049 (11.4) | 737 (10.8) | 129 (13.0) | 81 (15.4) | 63 (11.1) | 39 (14.1) |  |
| 1960 | 1253 (13.6) | 949 (13.9) | 124 (12.5) | 78 (14.8) | 57 (10.0) | 45 (16.2) |  |
| 1961 | 1290 (14.0) | 974 (14.2) | 148 (14.9) | 67 (12.7) | 68 (11.9) | 33 (11.9) |  |
| 1962 | 1346 (14.6) | 1018 (14.9) | 138 (13.9) | 77 (14.6) | 82 (14.4) | 31 (11.2) |  |
| 1963 | 1281 (13.9) | 948 (13.9) | 148 (14.9) | 70 (13.3) | 78 (13.7) | 37 (13.4) |  |
| 1964 | 1052 (11.4) | 793 (11.6) | 110 (11.1) | 51 (9.7) | 72 (12.6) | 26 (9.4) |  |
| **Gender, male (%)** | 4517 (49.1) | 3635 (53.2) | 380 (38.2) | 212 (40.3) | 217 (38.1) | 73 (26.4) | <0.001 |
| **Race/ethnicity (%)** |  |  |  |  |  |  | <0.001 |
| Black | 2776 (30.2) | 1936 (28.3) | 361 (36.3) | 204 (38.8) | 185 (32.5) | 90 (32.5) |  |
| Hispanic | 1810 (19.7) | 1338 (19.6) | 198 (19.9) | 97 (18.4) | 105 (18.4) | 72 (26.0) |  |
| Non-Black, Non-Hispanic (NBNH) | 4620 (50.2) | 3564 (52.1) | 436 (43.8) | 225 (42.8) | 280 (49.1) | 115 (41.5) |  |
| **Gender & race/ethnicity (%)** |  |  |  |  |  |  | <0.001 |
| Black woman | 1413 (15.3) | 893 (13.1) | 211 (21.2) | 121 (23.0) | 120 (21.1) | 68 (24.5) |  |
| Black man | 1363 (14.8) | 1043 (15.3) | 150 (15.1) | 83 (15.8) | 65 (11.4) | 22 (7.9) |  |
| Hispanic woman | 927 (10.1) | 641 (9.4) | 125 (12.6) | 53 (10.1) | 56 (9.8) | 52 (18.8) |  |
| Hispanic man | 883 (9.6) | 697 (10.2) | 73 (7.3) | 44 (8.4) | 49 (8.6) | 20 (7.2) |  |
| NBNH woman | 2349 (25.5) | 1669 (24.4) | 279 (28.0) | 140 (26.6) | 177 (31.1) | 84 (30.3) |  |
| NBNH man | 2271 (24.7) | 1895 (27.7) | 157 (15.8) | 85 (16.2) | 103 (18.1) | 31 (11.2) |  |
| **Country of Birth (%)** |  |  |  |  |  |  | 0.758 |
| US born | 8537 (92.7) | 6324 (92.5) | 933 (93.8) | 484 (92.0) | 537 (94.2) | 259 (93.5) |  |
| Born Outside the US | 668 (7.3) | 513 (7.5) | 62 (6.2) | 42 (8.0) | 33 (5.8) | 18 (6.5) |  |
| Missing | 1 (0.0) | 1 (0.0) | 0 (0.0) | 0 (0.0) | 0 (0.0) | 0 (0.0) |  |
| **State region (%)** |  |  |  |  |  |  | 0.007 |
| Northeast | 1718 (18.7) | 1289 (18.9) | 186 (18.7) | 82 (15.6) | 99 (17.4) | 62 (22.4) |  |
| North central | 2292 (24.9) | 1762 (25.8) | 234 (23.5) | 118 (22.4) | 119 (20.9) | 59 (21.3) |  |
| South | 3340 (36.3) | 2424 (35.4) | 357 (35.9) | 222 (42.2) | 242 (42.5) | 95 (34.3) |  |
| West | 1710 (18.6) | 1262 (18.5) | 201 (20.2) | 94 (17.9) | 100 (17.5) | 53 (19.1) |  |
| Missing | 146 (1.6) | 101 (1.5) | 17 (1.7) | 10 (1.9) | 10 (1.8) | 8 (2.9) |  |
| **Metropolitan  statistical area (MSA) (%)** |  |  |  |  |  |  | 0.034 |
| Not in MSA | 2621 (28.5) | 1950 (28.5) | 262 (26.3) | 159 (30.2) | 179 (31.4) | 71 (25.6) |  |
| MSA, not central city | 2469 (26.8) | 1887 (27.6) | 260 (26.1) | 119 (22.6) | 123 (21.6) | 80 (28.9) |  |
| MSA, central city not known | 2125 (23.1) | 1556 (22.8) | 242 (24.3) | 127 (24.1) | 134 (23.5) | 66 (23.8) |  |
| MSA, in central city | 1928 (20.9) | 1392 (20.4) | 225 (22.6) | 121 (23.0) | 132 (23.2) | 58 (20.9) |  |
| Missing | 63 (0.7) | 53 (0.8) | 6 (0.6) | 0 (0.0) | 2 (0.4) | 2 (0.7) |  |
| **Rural region (%)** |  |  |  |  |  |  | 0.207 |
| Rural | 1799 (19.5) | 1345 (19.7) | 173 (17.4) | 98 (18.6) | 131 (23.0) | 52 (18.8) |  |
| Urban | 7385 (80.2) | 5474 (80.1) | 820 (82.4) | 428 (81.4) | 439 (77.0) | 224 (80.9) |  |
| Missing | 22 (0.2) | 19 (0.3) | 2 (0.2) | 0 (0.0) | 0 (0.0) | 1 (0.4) |  |
| **Family size (%)** |  |  |  |  |  |  | 0.006 |
| 1 | 502 (5.5) | 385 (5.6) | 46 (4.6) | 31 (5.9) | 22 (3.9) | 18 (6.5) |  |
| 2 | 759 (8.2) | 512 (7.5) | 98 (9.8) | 57 (10.8) | 59 (10.4) | 33 (11.9) |  |
| 3-4 | 3284 (35.7) | 2458 (35.9) | 342 (34.4) | 182 (34.6) | 201 (35.3) | 101 (36.5) |  |
| 5+ | 4661 (50.6) | 3483 (50.9) | 509 (51.2) | 256 (48.7) | 288 (50.5) | 125 (45.1) |  |
| **Marital status (%)** |  |  |  |  |  |  | <0.001 |
| Single | 8303 (90.2) | 6252 (91.4) | 881 (88.5) | 461 (87.6) | 486 (85.3) | 223 (80.5) |  |
| Married | 749 (8.1) | 496 (7.3) | 92 (9.2) | 55 (10.5) | 65 (11.4) | 41 (14.8) |  |
| Other | 152 (1.7) | 89 (1.3) | 22 (2.2) | 9 (1.7) | 19 (3.3) | 13 (4.7) |  |
| Missing | 2 (0.0) | 1 (0.0) | 0 (0.0) | 1 (0.2) | 0 (0.0) | 0 (0.0) |  |
| **Number of Children (%)** |  |  |  |  |  |  | <0.001 |
| None | 8393 (91.2) | 6350 (92.9) | 879 (88.3) | 451 (85.7) | 486 (85.3) | 227 (81.9) |  |
| 1 | 607 (6.6) | 375 (5.5) | 85 (8.5) | 56 (10.6) | 61 (10.7) | 30 (10.8) |  |
| 2 or More | 206 (2.2) | 113 (1.7) | 31 (3.1) | 19 (3.6) | 23 (4.0) | 20 (7.2) |  |
| **Birth mother alive (%)** |  |  |  |  |  |  | <0.001 |
| No | 225 (2.4) | 158 (2.3) | 29 (2.9) | 16 (3.0) | 14 (2.5) | 8 (2.9) |  |
| Yes | 1596 (17.3) | 1112 (16.3) | 179 (18.0) | 114 (21.7) | 114 (20.0) | 77 (27.8) |  |
| Missing | 7385 (80.2) | 5568 (81.4) | 787 (79.1) | 396 (75.3) | 442 (77.5) | 192 (69.3) |  |
| **Birth father alive (%)** |  |  |  |  |  |  | <0.001 |
| No | 705 (7.7) | 491 (7.2) | 91 (9.1) | 56 (10.6) | 37 (6.5) | 30 (10.8) |  |
| Yes | 2507 (27.2) | 1716 (25.1) | 310 (31.2) | 179 (34.0) | 189 (33.2) | 113 (40.8) |  |
| Missing | 5994 (65.1) | 4631 (67.7) | 594 (59.7) | 291 (55.3) | 344 (60.4) | 134 (48.4) |  |
| **Highest grade completed (%)** |  |  |  |  |  |  | <0.001 |
| None | 8 (0.1) | 6 (0.1) | 1 (0.1) | 0 (0.0) | 0 (0.0) | 1 (0.4) |  |
| 1st - 5th Grade | 51 (0.6) | 36 (0.5) | 5 (0.5) | 5 (1.0) | 3 (0.5) | 2 (0.7) |  |
| 6th - 8th Grade | 1869 (20.3) | 1309 (19.1) | 229 (23.0) | 111 (21.1) | 143 (25.1) | 77 (27.8) |  |
| 9th - 12th Grade | 6319 (68.6) | 4685 (68.5) | 680 (68.3) | 386 (73.4) | 385 (67.5) | 183 (66.1) |  |
| 1st - 5th year of college | 954 (10.4) | 799 (11.7) | 79 (7.9) | 24 (4.6) | 38 (6.7) | 14 (5.1) |  |
| Missing | 5 (0.1) | 3 (0.0) | 1 (0.1) | 0 (0.0) | 1 (0.2) | 0 (0.0) |  |
| **Times Drank Alcohol in the Past Year, Under 17 [1980] (%)** |  |  |  |  |  |  | 0.027 |
| Never | 1287 (14.0) | 962 (14.1) | 137 (13.8) | 74 (14.1) | 82 (14.4) | 32 (11.6) |  |
| Once | 364 (4.0) | 264 (3.9) | 34 (3.4) | 17 (3.2) | 37 (6.5) | 12 (4.3) |  |
| 2-10 times | 995 (10.8) | 775 (11.3) | 106 (10.7) | 37 (7.0) | 50 (8.8) | 27 (9.7) |  |
| 11-50 times | 365 (4.0) | 280 (4.1) | 37 (3.7) | 18 (3.4) | 20 (3.5) | 10 (3.6) |  |
| 50+ times | 253 (2.7) | 190 (2.8) | 27 (2.7) | 17 (3.2) | 17 (3.0) | 2 (0.7) |  |
| 18 Years or Older | 4471 (48.6) | 3276 (47.9) | 482 (48.4) | 274 (52.1) | 281 (49.3) | 158 (57.0) |  |
| Missing | 1471 (16.0) | 1091 (16.0) | 172 (17.3) | 89 (16.9) | 83 (14.6) | 36 (13.0) |  |
| **Times Used Marijuana in  the Past Year [1980] (%)** |  |  |  |  |  |  | 0.004 |
| Never | 4712 (51.2) | 3585 (52.4) | 463 (46.5) | 266 (50.6) | 269 (47.2) | 129 (46.6) |  |
| Once | 767 (8.3) | 540 (7.9) | 84 (8.4) | 51 (9.7) | 59 (10.4) | 33 (11.9) |  |
| Twice | 370 (4.0) | 274 (4.0) | 41 (4.1) | 20 (3.8) | 21 (3.7) | 14 (5.1) |  |
| 3-5 times | 593 (6.4) | 424 (6.2) | 79 (7.9) | 34 (6.5) | 39 (6.8) | 17 (6.1) |  |
| 6-10 times | 403 (4.4) | 302 (4.4) | 52 (5.2) | 21 (4.0) | 15 (2.6) | 13 (4.7) |  |
| 11-50 times | 594 (6.5) | 451 (6.6) | 73 (7.3) | 28 (5.3) | 26 (4.6) | 16 (5.8) |  |
| More than 50 times | 1184 (12.9) | 858 (12.5) | 129 (13.0) | 72 (13.7) | 88 (15.4) | 37 (13.4) |  |
| Missing | 583 (6.3) | 404 (5.9) | 74 (7.4) | 34 (6.5) | 53 (9.3) | 18 (6.5) |  |
| **Times Used Hard  Drugs in the Past Year [1980] (%)** |  |  |  |  |  |  | 0.06 |
| Never | 7095 (77.1) | 5347 (78.2) | 724 (72.8) | 404 (76.8) | 418 (73.3) | 202 (72.9) |  |
| Once | 378 (4.1) | 272 (4.0) | 45 (4.5) | 20 (3.8) | 23 (4.0) | 18 (6.5) |  |
| Twice | 243 (2.6) | 180 (2.6) | 28 (2.8) | 13 (2.5) | 14 (2.5) | 8 (2.9) |  |
| 3-5 times | 324 (3.5) | 224 (3.3) | 44 (4.4) | 21 (4.0) | 21 (3.7) | 14 (5.1) |  |
| 6-10 times | 220 (2.4) | 166 (2.4) | 25 (2.5) | 9 (1.7) | 13 (2.3) | 7 (2.5) |  |
| 11-50 times | 219 (2.4) | 151 (2.2) | 34 (3.4) | 12 (2.3) | 17 (3.0) | 5 (1.8) |  |
| More than 50 times | 121 (1.3) | 80 (1.2) | 15 (1.5) | 9 (1.7) | 13 (2.3) | 4 (1.4) |  |
| Missing | 606 (6.6) | 418 (6.1) | 80 (8.0) | 38 (7.2) | 51 (8.9) | 19 (6.9) |  |
| **Ever "Stopped" by Police for  other than a minor traffic offense [1980] (%)** |  |  |  |  |  |  | 0.025 |
| No | 7296 (79.3) | 5479 (80.1) | 762 (76.6) | 411 (78.1) | 432 (75.8) | 212 (76.5) |  |
| Yes | 1582 (17.2) | 1132 (16.6) | 194 (19.5) | 93 (17.7) | 107 (18.8) | 56 (20.2) |  |
| Missing | 328 (3.6) | 227 (3.3) | 39 (3.9) | 22 (4.2) | 31 (5.4) | 9 (3.2) |  |
| **Ever Sentenced to a Correctional Institution [1980] (%)** |  |  |  |  |  |  | <0.001 |
| Never stopped by police | 7296 (79.3) | 5479 (80.1) | 762 (76.6) | 411 (78.1) | 432 (75.8) | 212 (76.5) |  |
| No | 1359 (14.8) | 988 (14.4) | 162 (16.3) | 77 (14.6) | 88 (15.4) | 44 (15.9) |  |
| Yes | 126 (1.4) | 68 (1.0) | 23 (2.3) | 15 (2.9) | 12 (2.1) | 8 (2.9) |  |
| Missing | 425 (4.6) | 303 (4.4) | 48 (4.8) | 23 (4.4) | 38 (6.7) | 13 (4.7) |  |

**Supplemental Table 2.2. Time Varying Demographic Factors, by Depressive Symptom Trajectory Group**

|  | **Overall** | **Low Depressive  Symptoms** | **Episodic,  Before Age 40** | **Episodic,  Age 40** | **Episodic,  Age 50** | **Persistent  Depressive Symptoms** | **p** |
| --- | --- | --- | --- | --- | --- | --- | --- |
| **n** | 9206 | 6838 | 995 | 526 | 570 | 277 |  |
| **State Region, age 20** |  |  |  |  |  |  | 0.002 |
| North Central | 2183 (24.3) | 1675 (25.1) | 223 (23.0) | 113 (21.9) | 115 (20.9) | 57 (21.3) |  |
| Northeast | 1659 (18.5) | 1239 (18.6) | 190 (19.6) | 81 (15.7) | 88 (16.0) | 61 (22.8) |  |
| South | 3381 (37.7) | 2452 (36.8) | 356 (36.7) | 226 (43.8) | 248 (45.0) | 99 (36.9) |  |
| West | 1744 (19.4) | 1297 (19.5) | 200 (20.6) | 96 (18.6) | 100 (18.1) | 51 (19.0) |  |
| **State Region, age 30** |  |  |  |  |  |  | 0.005 |
| North Central | 2017 (23.4) | 1540 (24.1) | 212 (22.5) | 99 (20.0) | 110 (20.7) | 56 (21.2) |  |
| Northeast | 1490 (17.3) | 1124 (17.6) | 162 (17.2) | 69 (14.0) | 82 (15.4) | 53 (20.1) |  |
| South | 3338 (38.8) | 2411 (37.8) | 360 (38.3) | 232 (47.0) | 235 (44.3) | 100 (37.9) |  |
| West | 1768 (20.5) | 1308 (20.5) | 207 (22.0) | 94 (19.0) | 104 (19.6) | 55 (20.8) |  |
| **State Region, age 40** |  |  |  |  |  |  | 0.007 |
| North Central | 1823 (23.6) | 1378 (24.4) | 189 (22.4) | 93 (20.0) | 104 (20.5) | 59 (24.7) |  |
| Northeast | 1201 (15.6) | 901 (15.9) | 128 (15.2) | 63 (13.5) | 65 (12.8) | 44 (18.4) |  |
| South | 3185 (41.3) | 2270 (40.1) | 354 (41.9) | 226 (48.6) | 243 (47.9) | 92 (38.5) |  |
| West | 1504 (19.5) | 1109 (19.6) | 173 (20.5) | 83 (17.8) | 95 (18.7) | 44 (18.4) |  |
| **State Region, age 50** |  |  |  |  |  |  | <0.001 |
| North Central | 1685 (23.3) | 1275 (24.3) | 179 (22.7) | 79 (18.4) | 104 (20.0) | 48 (21.4) |  |
| Northeast | 1088 (15.1) | 814 (15.5) | 114 (14.4) | 53 (12.3) | 69 (13.2) | 38 (17.0) |  |
| South | 3044 (42.2) | 2132 (40.6) | 338 (42.8) | 224 (52.1) | 254 (48.8) | 96 (42.9) |  |
| West | 1402 (19.4) | 1033 (19.7) | 159 (20.1) | 74 (17.2) | 94 (18.0) | 42 (18.8) |  |
|  |  |  |  |  |  |  |  |
| **Rural Region, age 20** |  |  |  |  |  |  | 0.001 |
| Rural | 1638 (17.8) | 1201 (17.6) | 161 (16.2) | 90 (17.1) | 127 (22.3) | 59 (21.3) |  |
| Urban | 6976 (75.8) | 5161 (75.5) | 786 (79.0) | 405 (77.0) | 415 (72.8) | 209 (75.5) |  |
| *Missing* | 592 (6.4) | 476 (7.0) | 48 (4.8) | 31 (5.9) | 28 (4.9) | 9 (3.2) |  |
| **Rural Region, age 30** |  |  |  |  |  |  | 0.025 |
| Rural | 1586 (17.2) | 1170 (17.1) | 152 (15.3) | 96 (18.3) | 119 (20.9) | 49 (17.7) |  |
| Urban | 6896 (74.9) | 5100 (74.6) | 781 (78.5) | 393 (74.7) | 409 (71.8) | 213 (76.9) |  |
| *Missing* | 724 (7.9) | 568 (8.3) | 62 (6.2) | 37 (7.0) | 42 (7.4) | 15 (5.4) |  |
| **Rural Region, age 40** |  |  |  |  |  |  | <0.001 |
| Rural | 2010 (21.8) | 1501 (22.0) | 183 (18.4) | 127 (24.1) | 140 (24.6) | 59 (21.3) |  |
| Urban | 5545 (60.2) | 4038 (59.1) | 650 (65.3) | 329 (62.5) | 355 (62.3) | 173 (62.5) |  |
| Unknown | 35 (0.4) | 26 (0.4) | 3 (0.3) | 3 (0.6) | 1 (0.2) | 2 (0.7) |  |
| *Missing* | 1616 (17.6) | 1273 (18.6) | 159 (16.0) | 67 (12.7) | 74 (13.0) | 43 (15.5) |  |
| **Rural Region, age 50** |  |  |  |  |  |  | <0.001 |
| Rural | 1494 (16.2) | 1087 (15.9) | 146 (14.7) | 95 (18.1) | 126 (22.1) | 40 (14.4) |  |
| Urban | 5434 (59.0) | 3957 (57.9) | 615 (61.8) | 314 (59.7) | 375 (65.8) | 173 (62.5) |  |
| Unknown | 289 (3.1) | 207 (3.0) | 30 (3.0) | 21 (4.0) | 20 (3.5) | 11 (4.0) |  |
| *Missing* | 1989 (21.6) | 1587 (23.2) | 204 (20.5) | 96 (18.3) | 49 (8.6) | 53 (19.1) |  |
|  |  |  |  |  |  |  |  |
| **Marital Status, Age 20** |  |  |  |  |  |  | <0.001 |
| Married | 1311 (14.2) | 882 (12.9) | 167 (16.8) | 90 (17.1) | 108 (18.9) | 64 (23.1) |  |
| Single | 7499 (81.5) | 5702 (83.4) | 776 (78.0) | 416 (79.1) | 418 (73.3) | 187 (67.5) |  |
| Other | 234 (2.5) | 134 (2.0) | 36 (3.6) | 12 (2.3) | 30 (5.3) | 22 (7.9) |  |
| *Missing* | 162 (1.8) | 120 (1.8) | 16 (1.6) | 8 (1.5) | 14 (2.5) | 4 (1.4) |  |
| **Marital Status, Age 30** |  |  |  |  |  |  | <0.001 |
| Married | 4565 (49.6) | 3611 (52.8) | 403 (40.5) | 228 (43.3) | 229 (40.2) | 94 (33.9) |  |
| Single | 2776 (30.2) | 1991 (29.1) | 338 (34.0) | 181 (34.4) | 182 (31.9) | 84 (30.3) |  |
| Other | 1393 (15.1) | 875 (12.8) | 208 (20.9) | 93 (17.7) | 128 (22.5) | 89 (32.1) |  |
| *Missing* | 472 (5.1) | 361 (5.3) | 46 (4.6) | 24 (4.6) | 31 (5.4) | 10 (3.6) |  |
| **Marital Status, Age 40** |  |  |  |  |  |  | <0.001 |
| Married | 4378 (47.6) | 3491 (51.1) | 385 (38.7) | 186 (35.4) | 243 (42.6) | 73 (26.4) |  |
| Single | 1461 (15.9) | 972 (14.2) | 192 (19.3) | 120 (22.8) | 120 (21.1) | 57 (20.6) |  |
| Other | 1955 (21.2) | 1257 (18.4) | 274 (27.5) | 164 (31.2) | 151 (26.5) | 109 (39.4) |  |
| *Missing* | 1412 (15.3) | 1118 (16.3) | 144 (14.5) | 56 (10.6) | 56 (9.8) | 38 (13.7) |  |
| **Marital Status, Age 50** |  |  |  |  |  |  | <0.001 |
| Married | 3898 (42.3) | 3108 (45.5) | 356 (35.8) | 152 (28.9) | 220 (38.6) | 62 (22.4) |  |
| Single | 1200 (13.0) | 788 (11.5) | 160 (16.1) | 98 (18.6) | 105 (18.4) | 49 (17.7) |  |
| Other | 2188 (23.8) | 1399 (20.5) | 286 (28.7) | 185 (35.2) | 203 (35.6) | 115 (41.5) |  |
| *Missing* | 1920 (20.9) | 1543 (22.6) | 193 (19.4) | 91 (17.3) | 42 (7.4) | 51 (18.4) |  |
|  |  |  |  |  |  |  |  |
| **Family Size, Age 20** |  |  |  |  |  |  | <0.001 |
| 1 | 1162 (12.6) | 922 (13.5) | 107 (10.8) | 53 (10.1) | 52 (9.1) | 28 (10.1) |  |
| 2 | 1104 (12.0) | 769 (11.2) | 122 (12.3) | 86 (16.3) | 79 (13.9) | 48 (17.3) |  |
| 3 or 4 | 3303 (35.9) | 2442 (35.7) | 350 (35.2) | 183 (34.8) | 223 (39.1) | 105 (37.9) |  |
| 5+ | 3476 (37.8) | 2585 (37.8) | 400 (40.2) | 197 (37.5) | 202 (35.4) | 92 (33.2) |  |
| *Missing* | 161 (1.7) | 120 (1.8) | 16 (1.6) | 7 (1.3) | 14 (2.5) | 4 (1.4) |  |
| **Family Size, Age 30** |  |  |  |  |  |  | <0.001 |
| 1 | 1617 (17.6) | 1218 (17.8) | 194 (19.5) | 82 (15.6) | 86 (15.1) | 37 (13.4) |  |
| 2 | 1744 (18.9) | 1358 (19.9) | 158 (15.9) | 91 (17.3) | 95 (16.7) | 42 (15.2) |  |
| 3 or 4 | 3854 (41.9) | 2852 (41.7) | 403 (40.5) | 235 (44.7) | 249 (43.7) | 115 (41.5) |  |
| 5+ | 1521 (16.5) | 1051 (15.4) | 194 (19.5) | 94 (17.9) | 109 (19.1) | 73 (26.4) |  |
| *Missing* | 470 (5.1) | 359 (5.3) | 46 (4.6) | 24 (4.6) | 31 (5.4) | 10 (3.6) |  |
| **Family Size, Age 40** |  |  |  |  |  |  | <0.001 |
| 1 | 1321 (14.3) | 899 (13.1) | 164 (16.5) | 109 (20.7) | 96 (16.8) | 53 (19.1) |  |
| 2 | 1355 (14.7) | 945 (13.8) | 174 (17.5) | 72 (13.7) | 114 (20.0) | 50 (18.1) |  |
| 3 or 4 | 3557 (38.6) | 2675 (39.1) | 359 (36.1) | 212 (40.3) | 219 (38.4) | 92 (33.2) |  |
| 5+ | 1563 (17.0) | 1202 (17.6) | 155 (15.6) | 77 (14.6) | 85 (14.9) | 44 (15.9) |  |
| *Missing* | 1410 (15.3) | 1117 (16.3) | 143 (14.4) | 56 (10.6) | 56 (9.8) | 38 (13.7) |  |
| **Family Size, Age 50** |  |  |  |  |  |  | <0.001 |
| 1 | 1686 (18.3) | 1101 (16.1) | 209 (21.0) | 150 (28.5) | 153 (26.8) | 73 (26.4) |  |
| 2 | 2087 (22.7) | 1476 (21.6) | 243 (24.4) | 130 (24.7) | 168 (29.5) | 70 (25.3) |  |
| 3 or 4 | 2795 (30.4) | 2159 (31.6) | 284 (28.5) | 119 (22.6) | 170 (29.8) | 63 (22.7) |  |
| 5+ | 719 (7.8) | 560 (8.2) | 66 (6.6) | 36 (6.8) | 37 (6.5) | 20 (7.2) |  |
| *Missing* | 1919 (20.8) | 1542 (22.6) | 193 (19.4) | 91 (17.3) | 42 (7.4) | 51 (18.4) |  |
| **Number of Children, Age 20** |  |  |  |  |  |  | <0.001 |
| None | 7641 (83.0) | 5870 (85.8) | 768 (77.2) | 397 (75.5) | 422 (74.0) | 184 (66.4) |  |
| 1 | 1054 (11.4) | 651 (9.5) | 159 (16.0) | 92 (17.5) | 96 (16.8) | 56 (20.2) |  |
| 2 or More | 350 (3.8) | 197 (2.9) | 52 (5.2) | 30 (5.7) | 38 (6.7) | 33 (11.9) |  |
| *Missing* | 161 (1.7) | 120 (1.8) | 16 (1.6) | 7 (1.3) | 14 (2.5) | 4 (1.4) |  |
| **Number of Children, Age 30** |  |  |  |  |  |  | <0.001 |
| None | 3766 (40.9) | 2871 (42.0) | 398 (40.0) | 201 (38.2) | 210 (36.8) | 86 (31.0) |  |
| 1 | 1739 (18.9) | 1310 (19.2) | 174 (17.5) | 96 (18.3) | 112 (19.6) | 47 (17.0) |  |
| 2 or More | 3214 (34.9) | 2285 (33.4) | 375 (37.7) | 200 (38.0) | 216 (37.9) | 138 (49.8) |  |
| *Missing* | 487 (5.3) | 372 (5.4) | 48 (4.8) | 29 (5.5) | 32 (5.6) | 6 (2.2) |  |
| **Number of Children, Age 40** |  |  |  |  |  |  | <0.001 |
| None | 1020 (11.1) | 709 (10.4) | 122 (12.3) | 66 (12.5) | 91 (16.0) | 32 (11.6) |  |
| 1 | 841 (9.1) | 582 (8.5) | 98 (9.8) | 57 (10.8) | 68 (11.9) | 36 (13.0) |  |
| 2 or More | 2994 (32.5) | 2073 (30.3) | 381 (38.3) | 194 (36.9) | 218 (38.2) | 128 (46.2) |  |
| *Missing* | 4351 (47.3) | 3474 (50.8) | 394 (39.6) | 209 (39.7) | 193 (33.9) | 81 (29.2) |  |
| **Number of Children, Age 50** |  |  |  |  |  |  | <0.001 |
| None | 603 (6.6) | 425 (6.2) | 66 (6.6) | 33 (6.3) | 58 (10.2) | 21 (7.6) |  |
| 1 | 614 (6.7) | 417 (6.1) | 74 (7.4) | 50 (9.5) | 49 (8.6) | 24 (8.7) |  |
| 2 or More | 2609 (28.3) | 1701 (24.9) | 372 (37.4) | 185 (35.2) | 225 (39.5) | 126 (45.5) |  |
| *Missing* | 5380 (58.4) | 4295 (62.8) | 483 (48.5) | 258 (49.0) | 238 (41.8) | 106 (38.3) |  |
| **Age of youngest child, Age 20 (median [IQR])** | 1.00  [0.00, 2.00] | 1.00  [0.00, 2.00] | 1.00  [0.00, 2.00] | 1.00  [0.00, 2.00] | 1.00  [0.00, 2.00] | 1.00  [0.00, 2.00] | 0.795 |
| **Age of youngest child, Age 30 (median [IQR])** | 3.00  [1.00, 6.00] | 3.00  [1.00, 5.00] | 3.00  [1.00, 6.00] | 4.00  [2.00, 7.00] | 4.00  [1.00, 7.00] | 4.00  [1.00, 7.00] | <0.001 |
| **Age of youngest child, Age 40 (median [IQR])** | 9.00  [5.00, 13.00] | 9.00  [5.00, 13.00] | 10.00  [5.00, 14.00] | 11.00  [6.00, 15.00] | 11.00  [7.00, 15.00] | 12.00  [8.00, 15.00] | <0.001 |
| **Age of youngest child, Age 50 (median [IQR])** | 17.00  [13.00, 21.00] | 17.00  [13.00, 20.00] | 17.00  [14.00, 22.00] | 18.00  [13.00, 23.00] | 18.00  [14.00, 23.00] | 20.00  [16.75, 24.00] | <0.001 |
| **Highest Level of  Education, Age 20** |  |  |  |  |  |  | <0.001 |
| Less than High School | 2212 (24.0) | 1374 (20.1) | 317 (31.9) | 209 (39.7) | 182 (31.9) | 130 (46.9) |  |
| High School Diploma | 6788 (73.7) | 5310 (77.7) | 661 (66.4) | 307 (58.4) | 369 (64.7) | 141 (50.9) |  |
| College Degree | 26 (0.3) | 23 (0.3) | 1 (0.1) | 1 (0.2) | 1 (0.2) | 0 (0.0) |  |
| *Missing* | 180 (2.0) | 131 (1.9) | 16 (1.6) | 9 (1.7) | 18 (3.2) | 6 (2.2) |  |
| **Highest Level of  Education, Age 30** |  |  |  |  |  |  | <0.001 |
| Less than High School | 1358 (14.8) | 817 (11.9) | 198 (19.9) | 129 (24.5) | 117 (20.5) | 97 (35.0) |  |
| High School Diploma | 5762 (62.6) | 4246 (62.1) | 657 (66.0) | 330 (62.7) | 372 (65.3) | 157 (56.7) |  |
| College Degree | 1604 (17.4) | 1412 (20.6) | 89 (8.9) | 42 (8.0) | 49 (8.6) | 12 (4.3) |  |
| *Missing* | 482 (5.2) | 363 (5.3) | 51 (5.1) | 25 (4.8) | 32 (5.6) | 11 (4.0) |  |
| **Highest Level of  Education, Age 40** |  |  |  |  |  |  | <0.001 |
| Less than High School | 899 (9.8) | 527 (7.7) | 126 (12.7) | 98 (18.6) | 83 (14.6) | 65 (23.5) |  |
| High School Diploma | 5239 (56.9) | 3766 (55.1) | 621 (62.4) | 323 (61.4) | 370 (64.9) | 159 (57.4) |  |
| College Degree | 1658 (18.0) | 1428 (20.9) | 105 (10.6) | 49 (9.3) | 61 (10.7) | 15 (5.4) |  |
| *Missing* | 1410 (15.3) | 1117 (16.3) | 143 (14.4) | 56 (10.6) | 56 (9.8) | 38 (13.7) |  |
| **Highest Level of  Education, Age 50** |  |  |  |  |  |  | <0.001 |
| Less than High School | 719 (7.8) | 414 (6.1) | 108 (10.9) | 79 (15.0) | 62 (10.9) | 56 (20.2) |  |
| High School Diploma | 4902 (53.2) | 3462 (50.6) | 579 (58.2) | 306 (58.2) | 401 (70.4) | 154 (55.6) |  |
| College Degree | 1666 (18.1) | 1420 (20.8) | 115 (11.6) | 50 (9.5) | 65 (11.4) | 16 (5.8) |  |
| *Missing* | 1919 (20.8) | 1542 (22.6) | 193 (19.4) | 91 (17.3) | 42 (7.4) | 51 (18.4) |  |

IQR, Interquartile Range

**Supplemental Table 2.3. Spearman Correlations Between Time-Varying Variables, Stratified by Depressive Symptom Trajectory Group**

| **Factor** | **Age 1** | **Age 2** | **Overall Cohort** | **Persistent Low Symptom** | **Episodic,  Before Age 40** | **Episodic, Around Age 40** | **Episodic, Around Age 50** | **Persistent High Symptom** |
| --- | --- | --- | --- | --- | --- | --- | --- | --- |
| State Region | 20 | 30 | 0.83 | 0.82 | 0.86 | 0.86 | 0.80 | 0.87 |
|  | 20 | 40 | 0.81 | 0.80 | 0.82 | 0.85 | 0.82 | 0.84 |
|  | 20 | 50 | 0.80 | 0.79 | 0.79 | 0.83 | 0.83 | 0.78 |
|  | 30 | 20 | 0.83 | 0.82 | 0.86 | 0.86 | 0.80 | 0.87 |
|  | 30 | 40 | 0.90 | 0.90 | 0.91 | 0.89 | 0.92 | 0.88 |
|  | 30 | 50 | 0.88 | 0.88 | 0.87 | 0.88 | 0.91 | 0.86 |
|  | 40 | 20 | 0.81 | 0.80 | 0.82 | 0.85 | 0.82 | 0.84 |
|  | 40 | 30 | 0.90 | 0.90 | 0.91 | 0.89 | 0.92 | 0.88 |
|  | 40 | 50 | 0.93 | 0.93 | 0.93 | 0.93 | 0.95 | 0.92 |
|  | 50 | 20 | 0.80 | 0.79 | 0.79 | 0.83 | 0.83 | 0.78 |
|  | 50 | 30 | 0.88 | 0.88 | 0.87 | 0.88 | 0.91 | 0.86 |
|  | 50 | 40 | 0.93 | 0.93 | 0.93 | 0.93 | 0.95 | 0.92 |
| Rural Region | 20 | 30 | 0.57 | 0.56 | 0.52 | 0.60 | 0.67 | 0.66 |
|  | 20 | 40 | 0.30 | 0.29 | 0.29 | 0.28 | 0.39 | 0.33 |
|  | 20 | 50 | 0.24 | 0.22 | 0.34 | 0.24 | 0.30 | 0.25 |
|  | 30 | 20 | 0.57 | 0.56 | 0.52 | 0.60 | 0.67 | 0.66 |
|  | 30 | 40 | 0.38 | 0.37 | 0.33 | 0.42 | 0.49 | 0.34 |
|  | 30 | 50 | 0.30 | 0.30 | 0.35 | 0.26 | 0.37 | 0.17 |
|  | 40 | 20 | 0.30 | 0.29 | 0.29 | 0.28 | 0.39 | 0.33 |
|  | 40 | 30 | 0.38 | 0.37 | 0.33 | 0.42 | 0.49 | 0.34 |
|  | 40 | 50 | 0.48 | 0.48 | 0.49 | 0.42 | 0.50 | 0.40 |
|  | 50 | 20 | 0.24 | 0.22 | 0.34 | 0.24 | 0.30 | 0.25 |
|  | 50 | 30 | 0.30 | 0.30 | 0.35 | 0.26 | 0.37 | 0.17 |
|  | 50 | 40 | 0.48 | 0.48 | 0.49 | 0.42 | 0.50 | 0.40 |
| Highest Grade Completed | 20 | 30 | 0.64 | 0.59 | 0.69 | 0.70 | 0.70 | 0.79 |
|  | 20 | 40 | 0.55 | 0.51 | 0.57 | 0.61 | 0.60 | 0.64 |
|  | 20 | 50 | 0.51 | 0.48 | 0.54 | 0.57 | 0.51 | 0.60 |
|  | 30 | 20 | 0.64 | 0.59 | 0.69 | 0.70 | 0.70 | 0.79 |
|  | 30 | 40 | 0.90 | 0.91 | 0.85 | 0.89 | 0.89 | 0.81 |
|  | 30 | 50 | 0.85 | 0.86 | 0.79 | 0.83 | 0.79 | 0.74 |
|  | 40 | 20 | 0.55 | 0.51 | 0.57 | 0.61 | 0.60 | 0.64 |
|  | 40 | 30 | 0.90 | 0.91 | 0.85 | 0.89 | 0.89 | 0.81 |
|  | 40 | 50 | 0.94 | 0.95 | 0.93 | 0.91 | 0.91 | 0.94 |
|  | 50 | 20 | 0.51 | 0.48 | 0.54 | 0.57 | 0.51 | 0.60 |
|  | 50 | 30 | 0.85 | 0.86 | 0.79 | 0.83 | 0.79 | 0.74 |
|  | 50 | 40 | 0.94 | 0.95 | 0.93 | 0.91 | 0.91 | 0.94 |
| Family Size | 20 | 30 | 0.07 | 0.07 | 0.12 | 0.12 | 0.06 | 0.05 |
|  | 20 | 40 | 0.07 | 0.07 | 0.08 | 0.02 | 0.09 | 0.05 |
|  | 20 | 50 | 0.08 | 0.09 | 0.06 | 0.05 | 0.05 | 0.03 |
|  | 30 | 20 | 0.07 | 0.07 | 0.12 | 0.12 | 0.06 | 0.05 |
|  | 30 | 40 | 0.41 | 0.41 | 0.42 | 0.39 | 0.41 | 0.45 |
|  | 30 | 50 | 0.15 | 0.14 | 0.25 | 0.20 | 0.13 | 0.29 |
|  | 40 | 20 | 0.07 | 0.07 | 0.08 | 0.02 | 0.09 | 0.05 |
|  | 40 | 30 | 0.41 | 0.41 | 0.42 | 0.39 | 0.41 | 0.45 |
|  | 40 | 50 | 0.50 | 0.51 | 0.47 | 0.40 | 0.42 | 0.52 |
|  | 50 | 20 | 0.08 | 0.09 | 0.06 | 0.05 | 0.05 | 0.03 |
|  | 50 | 30 | 0.15 | 0.14 | 0.25 | 0.20 | 0.13 | 0.29 |
|  | 50 | 40 | 0.50 | 0.51 | 0.47 | 0.40 | 0.42 | 0.52 |
| Marital Status | 20 | 30 | 0.11 | 0.12 | 0.12 | 0.11 | 0.16 | 0.02 |
|  | 20 | 40 | 0.04 | 0.05 | 0.02 | 0.07 | 0.09 | -0.08 |
|  | 20 | 50 | 0.02 | 0.04 | 0.03 | 0.03 | 0.04 | -0.12 |
|  | 30 | 20 | 0.11 | 0.12 | 0.12 | 0.11 | 0.16 | 0.02 |
|  | 30 | 40 | 0.38 | 0.39 | 0.27 | 0.33 | 0.39 | 0.29 |
|  | 30 | 50 | 0.31 | 0.32 | 0.21 | 0.18 | 0.29 | 0.25 |
|  | 40 | 20 | 0.04 | 0.05 | 0.02 | 0.07 | 0.09 | -0.08 |
|  | 40 | 30 | 0.38 | 0.39 | 0.27 | 0.33 | 0.39 | 0.29 |
|  | 40 | 50 | 0.55 | 0.55 | 0.51 | 0.52 | 0.45 | 0.49 |
|  | 50 | 20 | 0.02 | 0.04 | 0.03 | 0.03 | 0.04 | -0.12 |
|  | 50 | 30 | 0.31 | 0.32 | 0.21 | 0.18 | 0.29 | 0.25 |
|  | 50 | 40 | 0.55 | 0.55 | 0.51 | 0.52 | 0.45 | 0.49 |
| Number of Children | 20 | 30 | 0.34 | 0.32 | 0.35 | 0.36 | 0.48 | 0.37 |
|  | 20 | 40 | 0.22 | 0.20 | 0.21 | 0.25 | 0.31 | 0.31 |
|  | 20 | 50 | 0.30 | 0.27 | 0.28 | 0.33 | 0.42 | 0.35 |
|  | 30 | 20 | 0.34 | 0.32 | 0.35 | 0.36 | 0.48 | 0.37 |
|  | 30 | 40 | 0.63 | 0.61 | 0.65 | 0.67 | 0.72 | 0.72 |
|  | 30 | 50 | 0.68 | 0.67 | 0.67 | 0.67 | 0.75 | 0.81 |
|  | 40 | 20 | 0.22 | 0.20 | 0.21 | 0.25 | 0.31 | 0.31 |
|  | 40 | 30 | 0.63 | 0.61 | 0.65 | 0.67 | 0.72 | 0.72 |
|  | 40 | 50 | 0.93 | 0.94 | 0.87 | 0.91 | 0.89 | 0.90 |
|  | 50 | 20 | 0.30 | 0.27 | 0.28 | 0.33 | 0.42 | 0.35 |
|  | 50 | 30 | 0.68 | 0.67 | 0.67 | 0.67 | 0.75 | 0.81 |
|  | 50 | 40 | 0.93 | 0.94 | 0.87 | 0.91 | 0.89 | 0.90 |

**Supplemental Table 2.4 Health Status at Age 40 and 50, by Depressive Symptom Trajectory group**

|  | Overall | Low Depressive  Symptoms | Episodic,  Before Age 40 | Episodic,  Age 40 | | Episodic,  Age 50 | | Persistent Depressive Symptoms | | p | |
| --- | --- | --- | --- | --- | --- | --- | --- | --- | --- | --- | --- |
| n | 9206 | 6838 | 995 | 526 | | 570 | | 277 | |  | |
| **Health at 40 Module** |  |  |  |  | |  | |  | |  | |
| **Participated in  health at 40 Module (%)** | 8279 (89.9) | 6070 (88.8) | 911 (91.6) | 502 (95.4) | | 540 (94.7) | | 256 (92.4) | | <0.001 | |
| **Father Living (%)** |  |  |  |  | |  | |  | | <0.001 | |
| No | 3208 (38.7) | 2276 (37.5) | 386 (42.4) | 213 (42.4) | | 215 (39.8) | | 118 (46.1) | |  | |
| Yes | 4792 (57.9) | 3611 (59.5) | 484 (53.1) | 269 (53.6) | | 305 (56.5) | | 123 (48.0) | |  | |
| *Missing* | 279 (3.4) | 183 (3.0) | 41 (4.5) | 20 (4.0) | | 20 (3.7) | | 15 (5.9) | |  | |
| **Mother Living (%)** |  |  |  |  | |  | |  | | <0.001 | |
| No | 1663 (20.1) | 1154 (19.0) | 191 (21.0) | 124 (24.7) | | 114 (21.1) | | 80 (31.2) | |  | |
| Yes | 6520 (78.8) | 4850 (79.9) | 700 (76.8) | 374 (74.5) | | 422 (78.1) | | 174 (68.0) | |  | |
| *Missing* | 96 (1.2) | 66 (1.1) | 20 (2.2) | 4 (0.8) | | 4 (0.7) | | 2 (0.8) | |  | |
| **Doctor ever diagnosed  emotional, nervous,  or psychiatric problems (%)** | |  |  | |  | |  | |  | | <0.001 |
| No | 7642 (92.3) | 5861 (96.6) | 795 (87.3) | 369 (73.5) | | 471 (87.2) | | 146 (57.0) | |  | |
| Yes | 613 (7.4) | 192 (3.2) | 112 (12.3) | 132 (26.3) | | 68 (12.6) | | 109 (42.6) | |  | |
| *Missing* | 24 (0.3) | 17 (0.3) | 4 (0.4) | 1 (0.2) | | 1 (0.2) | | 1 (0.4) | |  | |
| **Self-reported depression,  excessive worry, or related trouble (%)** |  |  |  |  | |  | |  | | <0.001 | |
| No | 7158 (86.5) | 5687 (93.7) | 724 (79.5) | 258 (51.4) | | 393 (72.8) | | 96 (37.5) | |  | |
| Yes | 1088 (13.1) | 359 (5.9) | 184 (20.2) | 241 (48.0) | | 145 (26.9) | | 159 (62.1) | |  | |
| *Missing* | 33 (0.4) | 24 (0.4) | 3 (0.3) | 3 (0.6) | | 2 (0.4) | | 1 (0.4) | |  | |
| **Number of Health Conditions (%)** |  |  |  |  | |  | |  | | <0.001 | |
| No Conditions | 5653 (68.3) | 4409 (72.6) | 585 (64.2) | 255 (50.8) | | 296 (54.8) | | 108 (42.2) | |  | |
| 1 Condition | 1926 (23.3) | 1326 (21.8) | 211 (23.2) | 150 (29.9) | | 166 (30.7) | | 73 (28.5) | |  | |
| 2 or More Conditions | 700 (8.5) | 335 (5.5) | 115 (12.6) | 97 (19.3) | | 78 (14.4) | | 75 (29.3) | |  | |
| *Missing* | 54.84  [51.26, 56.51] | 55.26  [52.60, 56.58] | 53.80  [48.50, 55.91] | 50.47  [36.36, 56.18] | | 52.85  [46.05, 55.91] | | 47.50  [34.34, 54.78] | | <0.001 | |
| **SF-12 Physical Score (median [IQR])** | 55.74  [50.30, 57.92] | 56.77  [53.08, 58.73] | 53.24  [47.05, 57.16] | 42.45  [32.10, 50.13] | | 52.78  [44.97, 57.72] | | 36.89  [27.91, 47.28] | | <0.001 | |
| **SF-12 Mental Score (median [IQR])** |  |  |  |  | |  | |  | |  | |
| **Health at 50 Module** |  |  |  |  | |  | |  | |  | |
| **Participated in health at 50 Module (%)** | 7721 (83.9) | 5624 (82.2) | 846 (85.0) | 457 (86.9) | | 564 (98.9) | | 230 (83.0) | | <0.001 | |
| **Doctor ever diagnosed depression (%)** |  |  |  |  | |  | |  | | <0.001 | |
| No | 6459 (83.7) | 5148 (91.5) | 657 (77.7) | 297 (65.0) | | 271 (48.0) | | 86 (37.4) | |  | |
| Yes | 1243 (16.1) | 468 (8.3) | 187 (22.1) | 158 (34.6) | | 287 (50.9) | | 143 (62.2) | |  | |
| *Missing* | 19 (0.2) | 8 (0.1) | 2 (0.2) | 2 (0.4) | | 6 (1.1) | | 1 (0.4) | |  | |
| **Doctor ever diagnosed  other psychiatric conditions (%)** |  |  |  |  | |  | |  | | <0.001 | |
| No | 6899 (89.4) | 5354 (95.2) | 708 (83.7) | 315 (68.9) | | 407 (72.2) | | 115 (50.0) | |  | |
| Yes | 255 (3.3) | 88 (1.6) | 39 (4.6) | 23 (5.0) | | 86 (15.2) | | 19 (8.3) | |  | |
| *Missing* | 567 (7.3) | 182 (3.2) | 99 (11.7) | 119 (26.0) | | 71 (12.6) | | 96 (41.7) | |  | |
| **Number of Health Conditions (%)** |  |  |  |  | |  | |  | | <0.001 | |
| No Conditions | 4465 (57.8) | 3514 (62.5) | 439 (51.9) | 220 (48.1) | | 213 (37.8) | | 79 (34.3) | |  | |
| 1 Condition | 2103 (27.2) | 1463 (26.0) | 239 (28.3) | 142 (31.1) | | 182 (32.3) | | 77 (33.5) | |  | |
| 2 or More Conditions | 1148 (14.9) | 644 (11.5) | 166 (19.6) | 95 (20.8) | | 169 (30.0) | | 74 (32.2) | |  | |
| *Missing* | 5 (0.1) | 3 (0.1) | 2 (0.2) | 0 (0.0) | | 0 (0.0) | | 0 (0.0) | |  | |
| **SF-12 Physical Score  (median [IQR])** | 53.55 [46.63, 55.50] | 53.91 [49.99, 55.91] | 51.02 [41.31, 54.84] | 49.07 [33.87, 54.32] | | 41.65 [28.48, 54.08] | | 36.59  [26.78, 49.48] | | <0.001 | |
| **SF-12 Mental Score  (median [IQR])** | 55.87 [50.17, 58.00] | 57.36 [53.41, 58.75] | 53.68 [47.27, 57.89] | 50.41 [42.14, 56.57] | | 37.14 [29.94, 46.97] | | 38.80  [29.63, 48.57] | | <0.001 | |

IQR, Interquartile Range

**Supplemental Figure 2.1. Median Annual Work Hours between ages 18 to 59, by Depressive Symptom Trajectory Group**


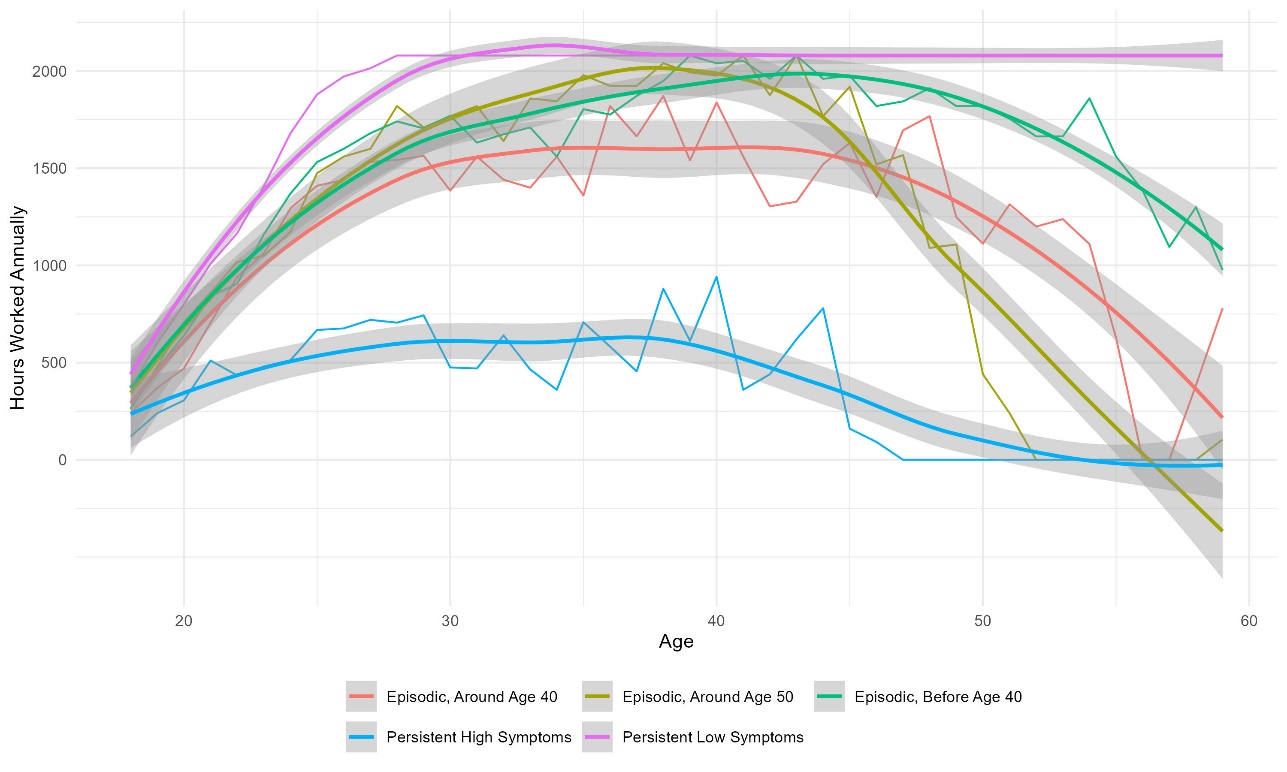


**Supplemental Figure 2.2. Average and Median Annual Employment Income between ages 18 to 61, by Depressive Symptom Trajectory**

**Panel A. Average Income**
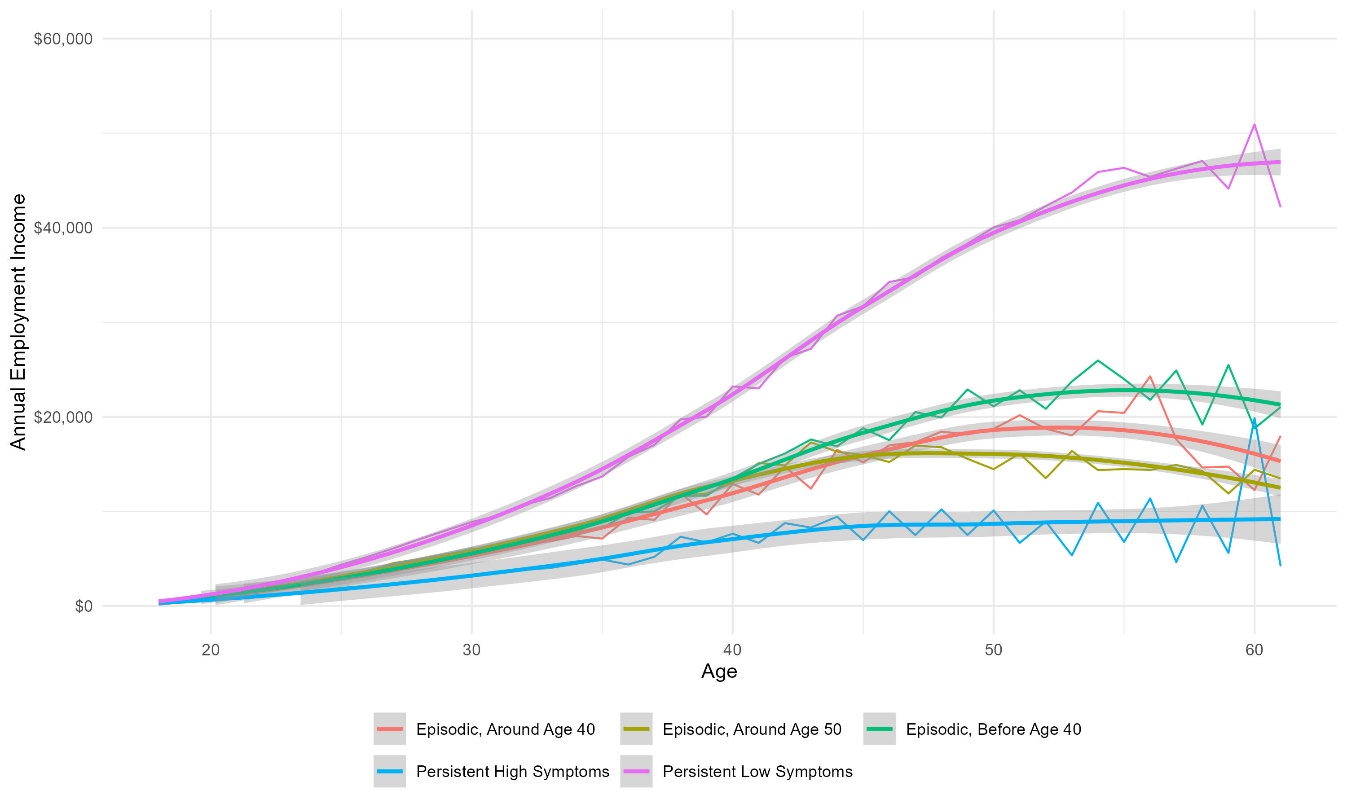


**Panel B. Median Income**

**
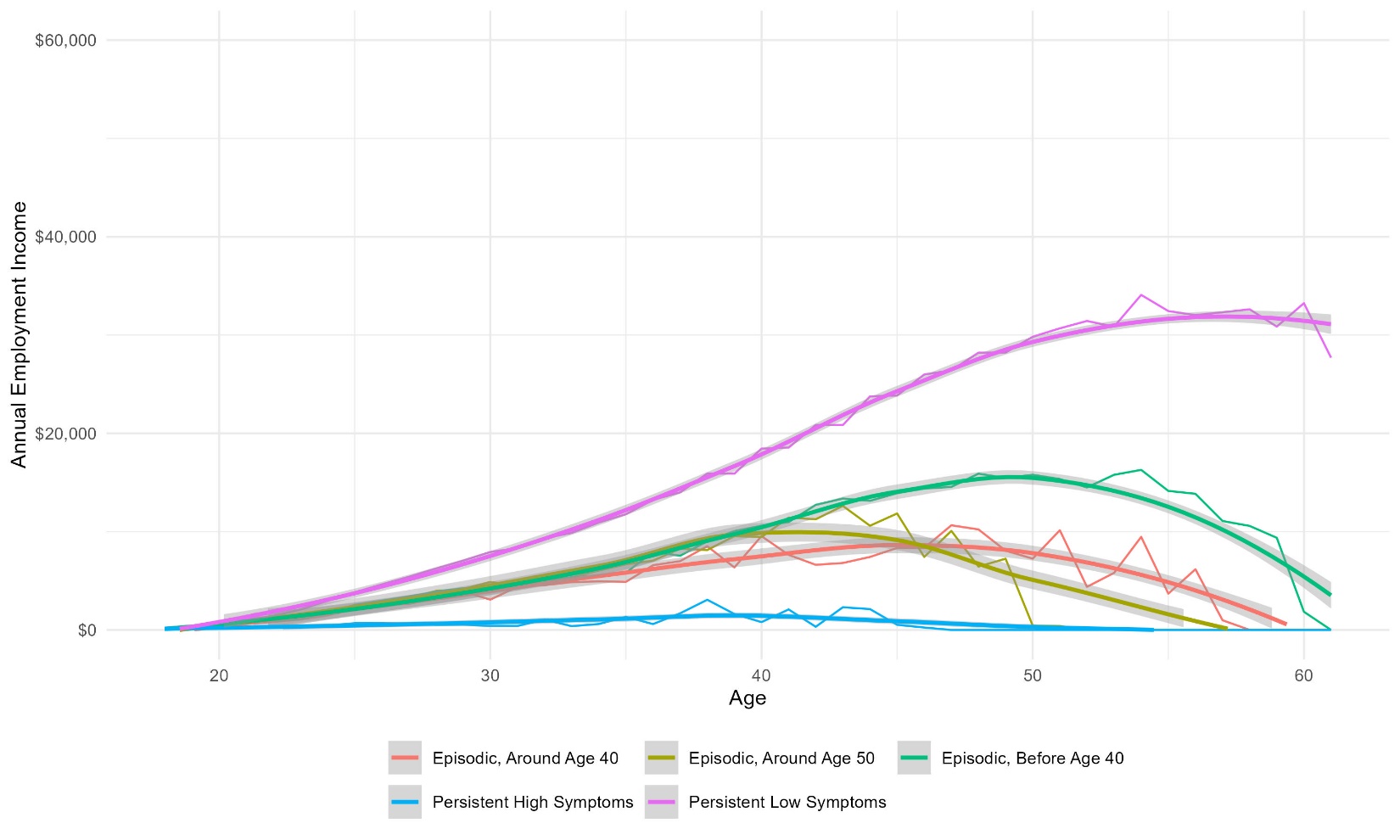
**

**Supplemental Figure 2.3. Median Annual Welfare Income between ages 18 to 61, by Depressive Symptom Trajectory**


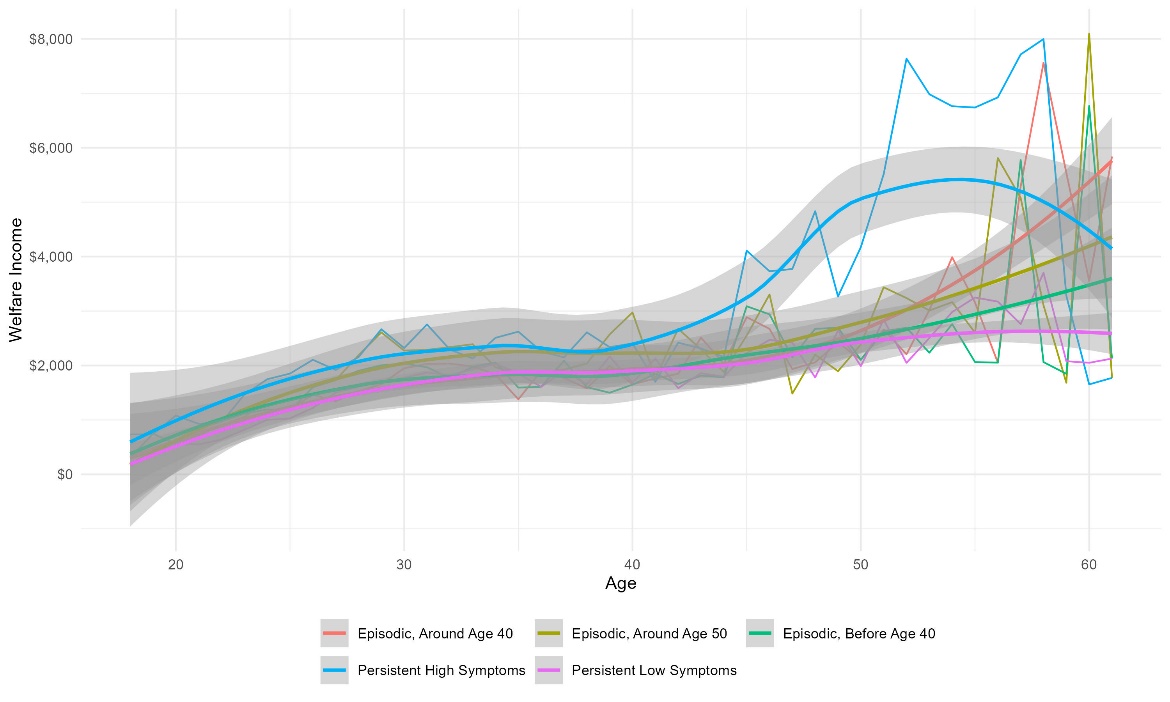

Supplement: Supplementary file 2 — Supplementary file2 (DOCX 762 KB) [file 127_2023_2547_MOESM2_ESM.docx]
